# A reliable, colloidal synthesis method of the orthorhombic chalcogenide perovskite, BaZrS_3_, and related ABS_3_ nanomaterials (A = Sr, Ba; B = Ti, Zr, Hf): a step forward for earth-abundant, functional materials†

**DOI:** 10.1039/d4sc06116k

**Published:** 2024-12-11

**Authors:** Daniel C. Hayes, Shubhanshu Agarwal, Kiruba Catherine Vincent, Izoduwa M. Aimiuwu, Apurva A. Pradhan, Madeleine C. Uible, Suzanne C. Bart, Rakesh Agrawal

**Affiliations:** a Davidson School of Chemical Engineering, Purdue University West Lafayette IN 47907 USA agrawalr@purdue.edu; b H.C. Brown Laboratory, James Tarpo Jr. and Margaret Tarpo Department of Chemistry, Purdue University West Lafayette IN 47907 USA

## Abstract

Recently, chalcogenide perovskites, of the form ABX_3_, where typically A = alkaline earth metals Ca, Sr, or Ba; B = group IV transition metals Zr or Hf; and X = chalcogens S or Se, have become of interest for their potential optoelectronic properties. In this work, we build upon recent studies and show a general synthesis protocol, involving the use of carbon disulfide insertion chemistry, to generate highly reactive precursors that can be used towards the colloidal synthesis of numerous ABS_3_ nanomaterials, including BaTiS_3_, BaZrS_3_, BaHfS_3_, α-SrZrS_3_ and α-SrHfS_3_. We overcome the shortcomings in the current literature where BaZrS_3_ nanoparticles are synthesized in separate phases *via* colloidal methods and lack a reproducible protocol for orthorhombic perovskite nanoparticles. We present a high-temperature, hot-injection method that reliably controls the formation of the colloidal BaZrS_3_ nanoparticles with the *Pnma* orthorhombic distorted perovskite structure. We show that the alternate phase, most notably denoted by its extra peaks in the pXRD pattern, is distinct from the distorted perovskite phase as it has a different bandgap value obtained *via* UV-vis measurements. We also show that the reaction byproducts, resulting from the use of oleylamine and CS_2_, have their own photoluminescence (PL) and their residual presence on the surface of the nanoparticles complicates the interpretation of PL from the nanoparticles. The utility of these nanomaterials is also assessed *via* the measurement of their absorption properties and in the form of highly stable colloidal inks for the fabrication of homogeneous, crack-free thin films of BaZrS_3_ nanoparticles.

## Abstract 2

Chalcogenide perovskites have become of interest for their increased stability, compared to their halide counterparts, and potential optoelectronic properties. A reliable colloidal method is presented here for BaZrS_3_ and related ABS_3_ nanomaterials.

## Graphical abstract

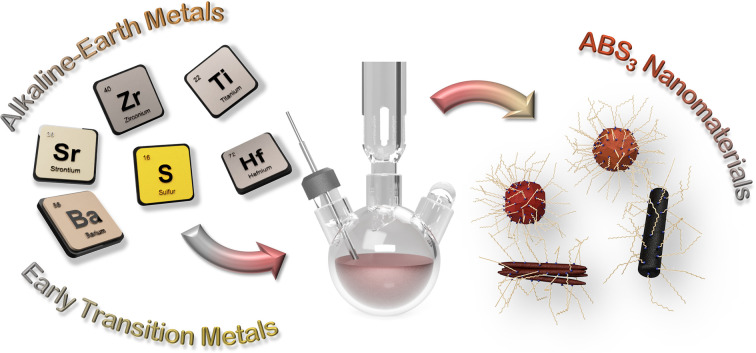

## Introduction

In the continuing pursuit of highly efficient renewable energy technologies, halide perovskites (HPs) have become ubiquitous in the semiconductor research community in just over the last decade. Their favorable optoelectronic properties for applications including light-emitting diodes (LEDs), photovoltaics (PVs), and photodetectors,^1^ demonstrate the wide utility of these materials. The word perovskite describes the crystal structure of the material and can be simplified to the formula ABX_3_, where, for HPs, A is a monovalent cation, typically Cs^+^, CH_3_NH_3_^+^ (methylammonium, MA), and/or CHNH_2_NH_2_^+^ (formadinium, FA); B is a divalent cation, typically Pb^2+^, Sn^2+^, and/or Ge^2+^; and X is a monovalent halide, typically Cl^−^, Br^−^, and/or I^−^. In terms of optical response, HPs have been shown to have remarkable luminescent properties. Lab-scale, single-junction solar cells of HPs have so far achieved a record power conversion efficiency of 26.7%.^2,3^ Aside from the enormous success HPs have been experiencing, they are also known to be susceptible to instabilities related to heat, moisture, and light exposure, among other factors,^4^ leading to concerns and uncertainty surrounding its anticipated scale-up to industrial scale and commercial usage. Much important work has been done to investigate ways at improving the stability and shelf life of HPs,^5^ but in order to meet the widely accepted standard warranty of 25 years for photovoltaic modules, much progress is still needed.

One approach to higher stability perovskites is through the use of an entirely different class of perovskites. Oxide perovskites—another class of perovskites which has an oxygen atom at the X-site—have been studied for several decades and show incredible stability, but their bandgaps are much too high for conventional optoelectronic applications, especially for PVs. Moving down the column from oxygen on the periodic table are the remaining chalcogens. Also capable of forming perovskites, this leads us to the chalcogenide perovskites (CPs). Aptly named, these perovskites take a divalent chalcogen, usually S^2−^ or Se^2−^, on the X site, while the A and B sites predominantly contain alkaline earth (AE) metals Ca^2+^, Sr^2+^, or Ba^2+^, and group IV transition metals (IVB) Ti^4+^, Zr^4+^, or Hf^4+^, respectively, to maintain charge balance and structural tolerance factors.^6^ Other combinations from different elements have been predicted and synthesized previously in the literature,^7–12^ but our focus will be on the AE and IVB metals.

More recently, CPs have gained a renewed interest for their potential optoelectronic applications, such as in tandem PV and LEDs, among others, due to their bandgap tunability between ∼1.5 and 2.4 eV. The main reason, arguably, is being a more stable alternative to HPs.^13,14^ These materials were first synthesized using solid-state techniques with typical protocols often requiring quite rigorous conditions (dwell times of several days and temperatures of at least 1100 °C).^15–17^ Niu *et al.* has investigated the stability of some of these materials in air revealing that under a steady heating rate, the perovskites BaZrS_3_ and SrZrS_3_ exhibit stability in air up until ∼650 °C—above which they each decompose into their respective oxygen-containing phases. These studies were performed on powders synthesized by solid-state techniques, requiring temperatures between 600 and 1100 °C for 60–100 hours.^18^ Experimental methods like these, however, are unsuitable for use in device fabrication, as subsequent layers in a device stack would not be able to withstand the extreme conditions without significant decomposition.

While much of the work on CPs is still focused on gaining a deeper understanding of the synthetic mechanisms and optoelectronic properties,^7,19–27^ there has been another push to fabricate these materials *via* low- to moderate-temperature pathways,^28–31^ including solution-processed approaches,^32–38^ to provide a much more suitable method for device fabrication. One notable difference between most of the traditional, high-temperature pathways and the low- to moderate-temperature pathways is the choice of precursors used. While chalcogenide perovskites are known to be highly stable materials,^14,18^ the fabrication of these materials requires a judicious choice of precursors that balances chemical reactivity and stability based on the synthesis conditions.^39^ With this in mind, we can group available precursors into highly stable or highly reactive precursors. These highly stable precursors usually consist of some combination of binary oxides, and/or the ABO_3_ ternary oxides. When high temperatures (>600 °C) are to be used, the highly stable precursors are often chosen as the experimental handling of these precursors is usually not burdensome. These metal oxides are extremely stable due to the high oxophilicity and hardness of the heavier AE and group IVB transition metals, Ti, Zr, and Hf. This causes these materials to readily form oxides when exposed to air or water at ambient conditions. As such, very large amounts of energy are needed to convert these oxides to their sulfide counterparts. It should be noted that very high temperatures are not always needed as recent studies show that the presence of a sufficiently effective “oxygen trap,” such as elemental boron or hafnium hydride, can be used to thermodynamically “trap” the oxygen and allow the precursors to react only with vicinal sulfur-containing species.^7,30^

The formation of ABS_3_ perovskites *via* low- to moderate-temperature methods, especially solution-based, must take a different approach. Due to the insufficient energy provided by these lower-temperature methods, the conversion of the stable oxide precursors into sulfides is highly unfavorable. Thus, the use of more reactive precursors must be adopted using, for example, precursors that contain M–C, M–N, or M–S bonds (where M = Ba, Sr, Hf, Zr, or Ti). Under a carefully prepared, inert environment, these precursors can decompose, usually under the presence of excess sulfur-containing species, and react to form the respective ABS_3_ materials.^32–37,40^ Due to the reactive nature of many of these types of precursors used, experimental preparation and handling of these materials is performed almost exclusively under inert environments, such as inert-filled gloveboxes, Schlenk line manifolds, and/or inert-filled furnaces. Under the proper conditions, even colloidal ABS_3_ nanomaterials (A = Sr, Ba; B = Ti, Zr, Hf) can be synthesized, previously thought to be unachievable at these lower temperatures due to the traditional methods used to prepare CPs.^14,32,33,39^ As a hallmark of solution-processing techniques, the use of colloidal nanocrystals provides a reliable way for the deposition of high-quality semiconductor materials as thin films.^41–46^ Additionally, colloidal nanocrystals can be influenced by the properties of surface ligands to change their luminescent yield and band alignment among other optoelectronic properties.^47,48^ These were both major motivating factors for our study.

In this work, we build upon the previous solution-processed literature on CPs and discuss improvements to the reported works on the colloidal synthesis of BaZrS_3_.^32,33^ The pioneering works by the Creutz and Hages groups detail the colloidal synthesis of BaZrS_3_ nanoparticles *via* the heat-up method where reactive precursors are added to oleylamine in a reaction vessel which is then heated to the reaction temperatures reported in the range of 275 °C to 365 °C. The major challenge of this method is that it generally results in nanoparticles that are a different polymorph of the Ba–Zr–S system (denoted hereafter as irregular phase BaZrS_3_, or IP-BZS) which can be distinguished by additional and/or broadened peaks in their measured diffraction patterns.^32,33,39^ In addition to the IP-BZS, however, the Creutz group was able to synthesize phase pure BaZrS_3_ nanoparticles, matching well to the *Pnma* orthorhombic distorted perovskite structure (denoted hereafter as standard phase BaZrS_3_, or SP-BZS), and attributed the formation of each of the two phases on high- and low-temperature reaction conditions. Their results suggested that the SP-BZS could only be formed at their highest reaction temperatures of 365 °C while the IP-BZS was formed under the majority of their other synthesis conditions where parameters like reaction temperature, concentration, and elemental ratios were varied. Regrettably, they indicate that the SP-BZS phase was difficult to replicate using their methods and that even repeat experiments under their standard reaction conditions at 365 °C would produce both phases. Here, we present a hot injection method using temperatures greater than 365 °C that reproducibly results in the colloidal synthesis of SP-BZS nanoparticles. Furthermore, through light absorption studies, we conclusively show that when compared to the SP-BZS nanoparticles, the IP-BZS nanoparticles indeed possess different properties. Another reported issue with the colloidal BaZrS_3_ nanoparticles concerns photoluminescence (PL). While Yang *et al.* discuss noticeable PL from their synthesized nanoparticles, the Creutz group reports to not see any PL from their nanoparticles.^32,33^ Here we present a detailed investigation regarding the associated PL issue and present evidence that any residual sulfur-containing reaction byproducts provide PL with an emission energy that is in the neighborhood of the reported PL from the bulk BaZrS_3_ and the as-synthesized nanoparticles in this study fail to show any significant PL at room temperature.

Through thorough and careful investigations, we have also developed a generalizable method for the synthesis of BaTiS_3_, BaHfS_3_, α-SrZrS_3,_ and α-SrHfS_3_ as colloidal nanomaterials from highly oxophilic precursors. α-SrHfS_3_ was only recently reported experimentally *via* solid-state methods,^24^ but previously theorized as an alternatively stable polymorph to the β-phase of SrHfS_3_.^14^ These methods also show the first proven colloidal syntheses of the Ba–Hf–S, Sr–Zr–S, and Sr–Hf–S systems. The synthesized BaZrS_3_ nanoparticles show a high level of colloidal stability for ink formulations, leading to coatings of high-quality, smooth films. Once films were coated, grain-coarsening experiments were also conducted, based on methods similar to those previously reported by our group,^30,31,35,36^ to study the effects of moderate-temperature sulfurizations.

## Results and discussion

### Synthesis of colloidal IP- and SP-BZS nanoparticles and observations made

Of the reports thus far on bottom-up colloidal synthesis of BaZrS_3_,^32,33^ the main commonality between the two is the use of highly reactive, organometallic precursors under moisture-free conditions (rigorously dried OLA and/or purified precursors). Specifically, Yang *et al.* utilized metal dithiocarbamate precursors for both Ba and Zr without any additional sulfur source. Zilevu *et al.* used a barium(trimethylsilyl)amide and a Zr(NMe_2_)_4_ species with a large excess of DETU and a Ba : Zr : S ratio of 1 : 2 : 60 for their standard protocol.

**Scheme 1:**
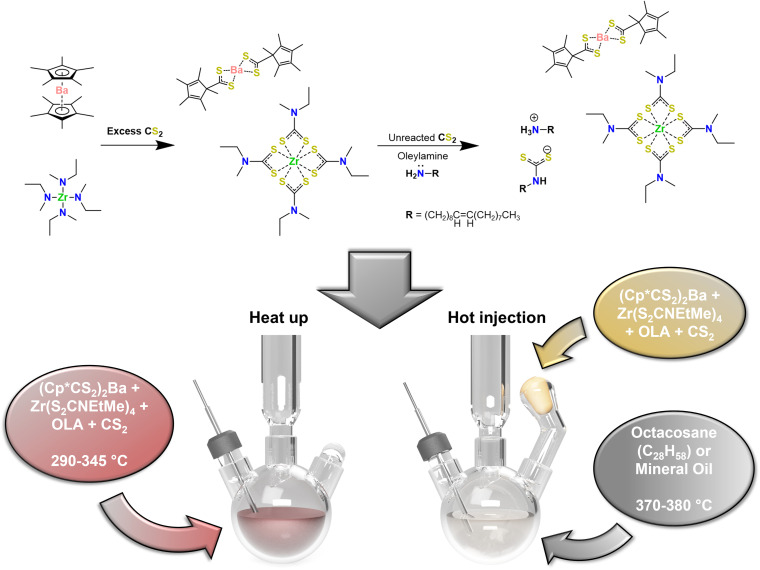
Precursor preparation and experimental protocol for BaZrS_3_ nanoparticle syntheses. Sr and Ti/Hf analogues were used in place of Ba and Zr, respectively, for BaTiS_3_, BaHfS_3_, SrZrS_3_, and SrHfS_3_ nanocrystal syntheses. Reaction ware setup is also illustrated (bottom).

In this work, our protocols also utilize highly reactive, organometallic precursors, beginning with Cp*_2_Ba and Zr(NEtMe)_4_ to react with CS_2_ to form (Cp*CS_2_)_2_Ba and Zr(S_2_CNEtMe)_4_.^36^ Because we used roughly 20 times the stoichiometric amount of sulfur, there was a large amount of unreacted CS_2_ that remained after combining all reactants in the reaction flask or Merlic adapter despite the exothermic nature of the reaction and high volatility of CS_2_ (b.p. = 46 °C). Upon addition of OLA to the excess CS_2_, the oleyldithiocarbamate–oleylammonium salt was formed, creating an additional sulfur source which was shown to be crucial for our syntheses. Shown in Scheme 1 is the standard preparation method used for synthesis of colloidal BaZrS_3_. After sufficient time following the addition of the OLA to the unreacted CS_2_, the reaction solvent solidified into an off-white/pale yellow solid, indicative of low-molecular-mass organic gelator behavior.^49^ The time between securing the reaction vessel inside the glovebox and setting up the reaction on the Schlenk line was usually sufficient for this to occur. Further details on the experimental setup are provided in the ESI.†

As described previously, most reactions were performed in two distinct ways: the one-pot, heat-up method—where all precursors and reactive materials were present in the reaction flask from the beginning—and the hot injection method—where precursors were added to the reaction flask *in situ* from the Merlic adapter after the heat-up of neat solvent at a prescribed temperature, after which, the system was allowed to dwell for a set time. During the heat-up method, many key observations were made: (1) the solid formed as a result of the OLA–CS_2_ product melted at ∼50–60 °C to form an opaque, milky, yellow-white solution. (2) Upon heating to ∼100–120 °C, vigorous yellow bubbling was observed, likely the result of some initial precursor decomposition and formation of an intermediate species. (3) Between ∼130 and 270 °C, a transparent, vibrant red to dark red solution was observed. By this point, it was clear that the reaction solution was entirely single-phase. (4) Between temperature readings of ∼270–290 °C—depending on how well the *in situ* thermocouple was in contact with the reaction solution—a second decomposition event occurred in which mild bubbling was observed. At this point, the solution turned into an opaque, dark red (indicating nucleation of nanoparticles), and condensation of sulfur-containing species (noted by its green and yellow color) was observed in the condenser. No noticeable changes were observed in the reaction solution based on color, aggregate formation, *etc.* during the dwell time, however, more sulfur-containing species were observed to condense along the condenser walls for longer reaction times. During the hot injection method, the OCA or mineral oil remained visibly unchanged, maintaining a colorless, transparent appearance, up to the prescribed heat-up temperature. Upon addition of the precursors, rapid release of gasses, heavy condensation of sulfur-containing species on the condenser walls, and rapid coloration of the reaction solution to dark red for reactions of the Ba–Zr–S system were observed.

**Fig. 1:**
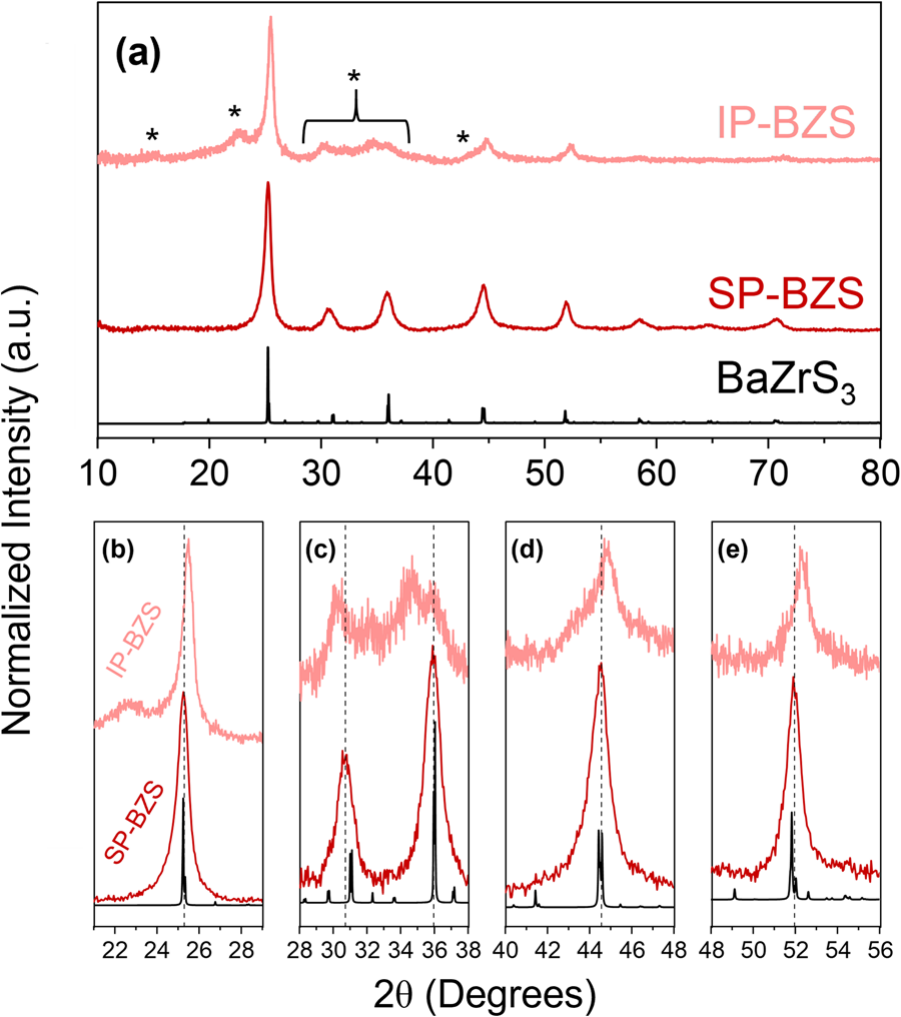
Structural characterization of synthesized BaZrS_3_ nanoparticles *via* pXRD of both obtained species shown in (a). The peaks labelled with * are the most easily identifiable additional peaks or differences that show in IP-BZS that are not present in SP-BZS. A comparison of the major peak positions between the two BaZrS_3_ phases synthesized in this work is shown in (b)–(e). In black is the BaZrS_3_ standard (ICSD# 23288), in dark red is SP-BZS, and in pink is IP-BZS. We note that in (c), several differences are present including peak shifts and additional peaks in IP-BZS.

**Fig. 2:**
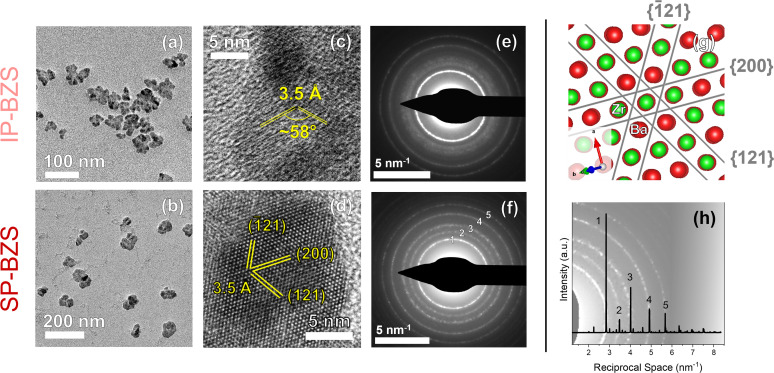
Images obtained from TEM analyses. Low-magnification images are shown in (a) and (b), high-resolution TEM (HRTEM) images with visible lattice planes are shown in (c) and (d), and SAED patterns are shown in (e) and (f) with data corresponding to IP-BZS on the top row and SP-BZS on the bottom row. (g) Shows a representation of the lattice planes in (d) visualized using VESTA^50^ from the [01̄2] zone axis. We should note that other orientations, such as from the [2̄10] zone axis with the (121), (121̄), and (002) lattice planes, are also equally plausible given the same lattice spacings and dihedral angles of these planes with those shown in (d) and (g), but show one example here for the sake of brevity. (h) Shows the BaZrS_3_ pXRD standard (ICSD# 23288), converted to reciprocal space, overlaid on top of the SAED pattern of (f) to show the agreement between the two.

Our heat-up results are similar to those by Zilevu *et al.*^33^ Below reaction temperatures of ∼330 °C in OLA, IP-BZS nanoparticles are always synthesized. However, when rapidly heated to the highest temperatures in the technical grade OLA (measured at ∼345 °C in our system), SP-BZS nanoparticles are randomly synthesized in a few runs with IP-BZS resulting from most of the runs. Even heat up in OCA or mineral oil to reaction temperatures approaching 380 °C resulted in IP-BZS. This indicated that the high reaction temperatures are not solely responsible for the synthesis of SP-BZS. On the other hand, hot injections at temperatures greater than 365 °C reproducibly resulted in the synthesis of SP-BZS as shown by the pXRD patterns shown in Fig. 1. TEM images along with selected area electron diffraction (SAED) patterns obtained for the two phases are also shown in Fig. 2.

Shown here, we obtain two distinct diffraction patterns for the synthesized nanoparticles, as seen in the pXRD and SAED patterns. One result appears to match much more closely to the given BaZrS_3_ standard (the SP-BZS), while the IP-BZS appears to contain additional, distinguishable crystal planes, shown by the additional diffraction peaks and rings in the pXRD and SAED patterns, respectively. For the IP-BZS, some slight peak shifting can also be observed for the peaks near 25°, 45°, and 52°, shown in Fig. 1b through 1e, indicating slight shrinkage for the irregular phase lattice. Interestingly though, each 2*θ* shift in (b), (d), and (e) is approximately equivalent (Δ*θ*_b_ ≈ Δ*θ*_d_ ≈ Δ*θ*_e_ ≈ 0.3°). Under most cases, when the unit cell volume increases (or decreases) by a certain amount, the shifts become proportionally larger as the 2*θ* position increases as described by Bragg's law for adjacent lattice planes, *λ* = 2*d* sin *θ*. Thus, we should expect Δ*θ*_b_ < Δ*θ*_d_ < Δ*θ*_e_, but this is not what is observed, suggesting that the IP-BZS crystal structure is different from the SP-BZS structure in more ways than simple lattice parameter distortions. It should also be noted that the IP-BZS tended to have some slight batch-to-batch variation, such as variable distinctiveness of certain peaks at 15°, 22.5° and in the region between ∼28 and 38° in the pXRD patterns. This is also evident in the SAED patterns shown in Fig. 2e and S6† where the region just outside the brightest diffraction ring, corresponding to the ∼28–38° region in the pXRD pattern, is not a distinct ring (or rings) but rather a collection of several spots within a confined radius from the center beam. Although unable to definitively conclude with the current data, this either indicates an increased number of reflections in this region for IP-BZS, with incomplete particle statistics leading to the incomplete rings, and/or the presence of an assortment of different phases rather than one singular phase. More detailed structural characterization is needed for a more complete understanding of the IP-BZS crystal structure.

The wide-view TEM images show that the BZS nanoparticles are somewhat irregular in shape. It was suggested in previous literature that this could be due to the coalescence of multiple smaller domains into one larger nanocrystal.^32^ The HRTEM images display lattice fringes consistent with what we should expect for BaZrS_3_ based on the pXRD and SAED data where the most prominent fringes correspond to the main diffraction peak at ∼25° 2*θ*. To help identify the lattice fringes, a fast Fourier transform (FFT) was performed on each image and analyzed, shown in Fig. S4.† While these differences are present in their crystal structure, both BZS species show a homogeneous composition of Ba, Zr, and S across the nanoparticles, as shown by STEM-EDX data in Fig. S5;† however, as shown later in the section titled Film fabrication and optoelectronic properties of ABS_3_ compounds, the IP-BZS nanoparticles are shown to have a lower bandgap than the SP-BZS and are therefore composed of a distinctly different phase as compared to the orthorhombic distorted perovskite structure of BaZrS_3_.

Next, we will discuss our investigations into the formation of the different phases and how we developed methods to reliably control each, notably the SP-BZS phase, which has eluded previous researchers.^32,33^

### Phase control of colloidal BaZrS_3_ nanoparticles

**Fig. 3:**
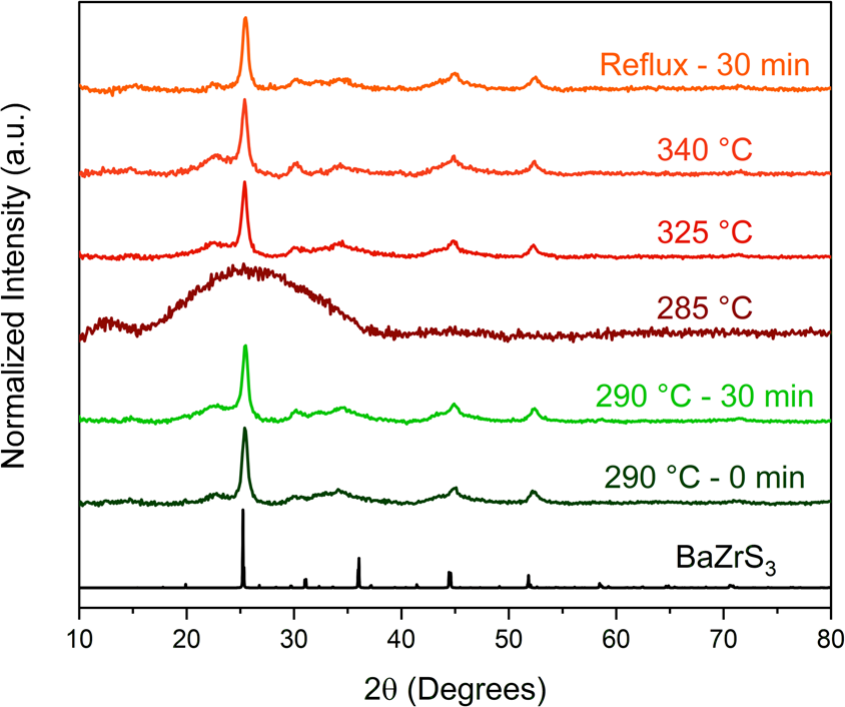
pXRD patterns of samples taken during an aliquot study of a BaZrS_3_ nanoparticle syntheses. Each reaction here followed the standard protocol for the heat-up method in OLA. Aliquots were taken at the indicated temperature during heat up and/or time indicated. The top four samples (285 °C to reflux) are from one experiment and the 290 °C samples are from a separate experiment. The 285 °C sample was taken just before nucleation was visually observed to occur. The data here indicates that the nanoparticles have a very quick nucleation and growth stage as no trend is observed for the FWHM values of the peak at ∼25° between each sample (excluding the 285 °C sample; see Table 1).

To gain insight into the formation of the two phases, a time–temperature study was conducted where aliquot samples were taken from near the nucleation point (∼270–290 °C) up until the reflux temperature of ∼345 °C of the technical grade OLA for the heat-up case and the results are shown in Fig. 3. In the sample collected at ∼285 °C, just before nucleation was observed, no significant particle formation or growth were seen as indicated by the pXRD pattern (Fig. 3). However, in the sample collected just after nucleation was observed to occur (as the reaction temperature reached 290 °C), significant and rapid nucleation and growth of the particles are observed, indicated by the well-defined pXRD pattern as compared to the other aliquot samples (Fig. 3). Interestingly, FWHM at 25° 2*θ* for all the samples including the one with the no dwell time at 290 °C and those at higher temperatures from a separate run are of similar values, indicating the rapid nucleation and brief growth phase of the nanoparticles (Table 1). The heat-up reaction temperature was further raised to 380 °C by using an equimolar solution of OLA and octacosane (OCA)—a heavy, linear alkane (m.p. = 65 °C, b.p. = 432 °C). While the waxy nature of OCA prevented adequate washing of the synthesized nanoparticles and thus collection of an interpretable pXRD pattern, a SAED pattern obtained from TEM measurements revealed the resulting product was still in the IP-BZS phase as shown in Fig. S6.† These observations show that during heat-up, the nucleation and growth kinetics of the IP-BZS phase are favorable at temperatures in the neighborhood of 290 °C, and the formed nanoparticles with this phase persist for the remaining duration even up to the relatively higher temperatures of 380 °C.

**Table 1:** FWHM of 25° peak in Fig. 3

Sample	FWHM (°)
Reflux – 30 min	0.658
340 °C	0.858
325 °C	0.757
285 °C	12.3
290 °C – 30 min	0.768
290 °C – 0 min	0.669

Injection of the precursors, as outlined in Scheme 1, at temperatures greater than 365 °C reliably led to the synthesis of SP-BZS nanoparticles with the *Pnma* orthorhombic distorted perovskite structure (Fig. 1). On the other hand, nanoparticles synthesized *via* the hot injection method at lower temperatures, such as in OLA at 345 °C, still contained IP-BZS nanoparticles (Run 20 in Table S1†). This indicates that the nucleation and growth of the orthorhombic distorted perovskite phase are favored—while that of IP-BZS are suppressed at higher temperatures (*T* > 365 °C). Furthermore, the rapid decomposition of precursors and the resulting molecules at these higher temperatures which is made possible by the hot injection procedure could favor the formation of SP-BZS. It is worth noting that due to the difficulties of post-reaction work-up when using OCA, most hot injection studies were conducted using high-purity mineral oil, a liquid at room temperature, with a boiling point of at least 375 °C. This allowed us to properly wash the nanoparticles post-synthesis for characterization and further use.

In addition to Cp*_2_Ba, Zr(NEtMe)_4,_ and CS_2_ in OLA and/or mineral oil, several other reactants and reaction conditions were used which are summarized in Table S1 in the ESI.† Some notable results in conjunction with Cp*_2_Ba and Zr(NEtMe)_4_ precursors are: (1) hot injections at lower temperatures resulted in the presence of IP-BZS in the final reaction product (Fig. S7†). (2) Replacement of CS_2_ with either MePT or DDT as the sulfur source in the heat-up reaction flask resulted in the formation of amorphous materials whereas replacement with DETU resulted in the IP-BZS. The use of Ba and Zr halide and acac precursors in conjunction with CS_2_ provided amorphous material along with some ZrO_2_. Additional pXRD data is also shown in Fig. S7† from some of these additional experimental conditions.

We would like to note that another common technique used for the structural identification of materials is Raman spectroscopy—the results of which we do not include in this main discussion. Attempted collection of Raman spectra from these nanomaterials proved inconclusive as our Raman detector would become over-saturated with signal due to what we concluded was the luminescence of some difficult-to-remove byproduct. Our Raman spectrometer has an excitation wavelength of 633 nm, which is well within the excitation emission of the notable reaction byproducts (see the Film fabrication and optoelectronic properties of ABS_3_ compounds section for more details). Under certain reaction conditions—both shorter times (>30 min) and lower temperatures (<300 °C)—we were able to detect a discernable Raman signal which we have shown in Fig. S8.† The Raman signal we obtain is much different than that previously reported for the *Pnma* orthorhombic perovskite BaZrS_3_ which we attribute to the structural differences that may be present between the IP- and SP-BZS phases. However, we must also consider the possibility of forming Ruddlesden–Popper (RP) phases when we mention IP-BZS. While RP phases are less thermodynamically stable than the perovskite phase, colloidal nanomaterial syntheses have the unique ability to stabilize materials in metastable phases,^51^ indicating formation of RP phases of the Ba–Zr–S system is a possibility that may be affecting some of our results. However, analysis of the Raman spectra and the pXRD pattern for the same sample in Fig. S8† suggests that the RP phase may not be forming and the IP-BZS phase has a different structure and/or stoichiometry from BaZrS_3_ and other known RP phases. The mechanism(s) behind phase control in solution-phase growth of inorganic nanocrystals is (are) still not well understood^52^ making the Ba–Zr–S system, among other material systems, a great candidate for further study in this regard.

### Preparation of additional colloidal ABS_3_ nanomaterials

**Fig. 4:**
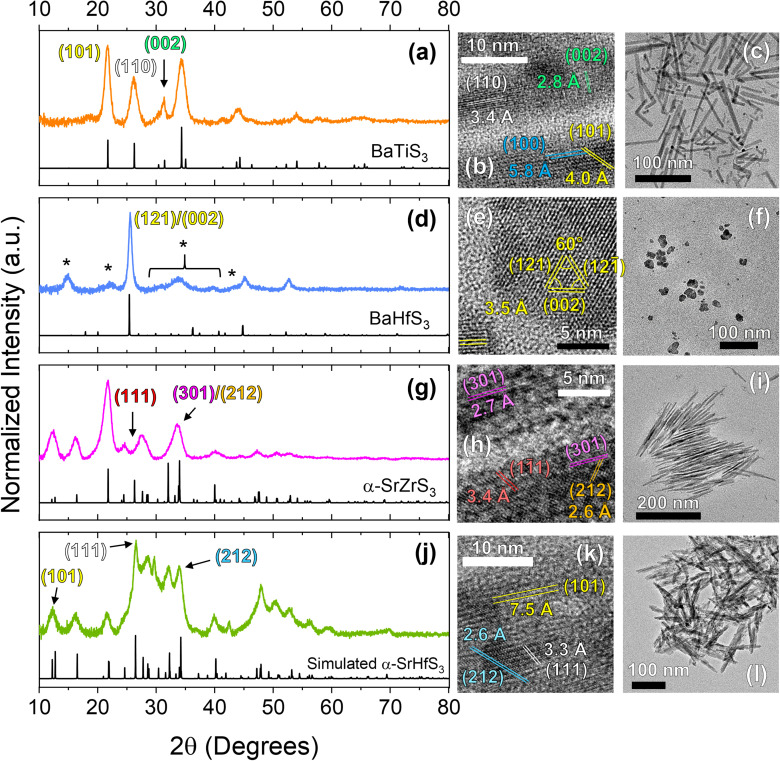
Structural characterization of the other colloidal ABS_3_ compounds investigated in this study. The rows from the top to the bottom show data corresponding to BaTiS_3_ (a–c), BaHfS_3_ (d–f), α-SrZrS_3_ (g–i), and α-SrHfS_3_ (j–l) respectively with the left, middle, and right-side columns corresponding to pXRD, HRTEM, and wide-view TEM data respectively. * in (d) are to mark additional peaks not corresponding to the given BaHfS_3_ standard. XRD standards for BaTiS_3_, BaHfS_3_, and α-SrZrS_3_ are ICSD# 616087, ICSD# 615918, and ICSD# 154103, respectively. The standard for α-SrHfS_3_ was simulated with a low level of structure optimization using VESTA^50^ software. As such, the generated structure can likely undergo further optimization for more accurate unit cell parameters. We note that the standards shown in this figure are simulated as a bulk, non-oriented powder pattern, so changes in certain relative intensities for species with oriented growth like BaTiS_3_, SrZrS_3_, and SrHfS_3_ are to be expected.

In addition to BaZrS_3_, we also investigated the applicability of the heat-up protocol to other colloidal ABS_3_ materials which was shown to work for the Ba–Ti–S, Ba–Hf–S, Sr–Zr–S, and Sr–Hf–S systems. By substituting the metal precursors with Ti(NMe_2_)_4_, Hf(NEtMe)_4_, and (Cp^iPr3^)_2_Sr where appropriate, we were able to synthesize BaTiS_3_ nanorods, and for the first time, colloidal Ba–Hf–S, SrZrS_3_, and SrHfS_3_ nanocrystals. We give the Ba–Hf–S material this label as the synthesized colloidal nanoparticles do not fully match the BaHfS_3_ pXRD standard and we have not been able to determine their definitive identity and composition *via* other means (further discussion to follow). We note that under the conditions used in this study, BaTiS_3_ grew as the BaNiO_3_-type hexagonal phase, and SrZrS_3_ and SrHfS_3_ grew as the NH_4_CdCl_3_-type α-phase or needle-like phase. While bulk SrZrS_3_ and SrHfS_3_ have been reported to form in the distorted perovskite phase, it has been shown that much higher temperatures are needed to achieve this—various reports using solid-state techniques indicate temperatures between 700 and 980 °C are necessary^14,24^—which is much too high for traditional colloidal synthesis methods. Shown in Fig. 4 is a structural and morphological analysis of these four species *via* pXRD and TEM techniques. Analysis of the HRTEM images shown below were also performed *via* FFTs which are also shown in Fig. S4.† STEM-EDX images are also shown in Fig. S9.† From these figures, we can see that BaTiS_3_ grows into nanorod structures, similar to what has been seen previously,^34,40^ Ba–Hf–S grows into pseudospherical nanoparticles, and SrZrS_3_ and SrHfS_3_ grow into needle-like structures (with SrZrS_3_ growing into considerably more defined needle-like structures than SrHfS_3_).

Interestingly, a previous study on colloidal BaTiS_3_ has shown that nanorods can be formed *via* a hot-injection method, while pseudospherical nanoparticles can be grown from a one-pot, heat-up method when using diethylthiourea (DETU) as the sulfur source.^34^ Our results show that the one-pot, heat-up method results in nanorod formation, although at a higher rate of polydispersity of nanorod dimensions (hot-injection methods typically lead to lower polydispersity due to a much more well-defined nucleation stage). We are able to form rods from a heat-up procedure likely as a result of the different precursors used, namely the sulfur source (oleyldithiocarbamates instead of DETU). During heat-up, the free oleyldithiocarbamate may decompose at a rate different than the DETU, impacting the nucleation and growth events. If the rate of availability of sulfur is low, the nanocrystals could attain a higher degree of dimensionality as higher-energy facets consume the limited, available sulfur more quickly than lower-energy facets.

The synthesized Ba–Hf–S nanoparticles are shown by comparison of the experimental pattern to the standard pattern to also exhibit many additional reflections as seen by the additional peaks, much like IP-BZS. While somewhat different in peak intensities, several of these peaks appear in similar positions to that of the IP-BZS, especially at 15°, 22.5°, and the shoulder at ∼44°. This, likely being due to the very similar ionic radii that zirconium and hafnium have—not to mention the standard diffraction patterns of BaZrS_3_ and BaHfS_3_ are very similar—leads us to believe that these Ba–Hf–S nanoparticles are also forming as a similar, irregular phase.

For the Sr–Zr–S system, we substituted Cp*_2_Ba for (Cp^iPr3^)_2_Sr and again followed the standard heat-up protocol to synthesize α-SrZrS_3_. Here, we see the formation of very thin nanoneedles, crystallizing in the aptly named needle-like phase. Similarly, for SrHfS_3_, we also substituted Zr(NEtMe)_4_ with Hf(NEtMe)_4_ to synthesize α-SrHfS_3_. SrHfS_3_ has only recently been reported experimentally in the α phase, but has been suggested to be possible in previous literature due to the chemical similarities between Zr and Hf.^14,24^ More success was seen for SrHfS_3_ when performing reactions at higher concentrations (≥0.16 M; at least 4× the concentration of the standard protocol). Efforts were also made to try and synthesize the standard-phase BaHfS_3_ as well as the β-SrZrS_3_ perovskite structure utilizing the same hot injection procedure using the Merlic adapter with high-temperature mineral oil. Unfortunately, our attempts were unsuccessful in forming the standard orthorhombic perovskite phase (shown in Fig. S10†), possibly due to the need for even higher temperatures not suited for the current colloidal synthesis method.

The synthesis of additional ABS_3_ species were also investigated but proved to be unsuccessful in this work. We looked into additional ABS_3_ species like Sr_*x*_TiS_3_, CaZrS_3_, and CaHfS_3_—the latter two also known to exist in the perovskite phase in the literature—but were unsuccessful in forming the ternary phase of any of these compounds. Our attempts at these compounds are summarized in Table S2.† While our efforts to synthesize the orthorhombic distorted perovskite phase of BaHfS_3_, SrZrS_3_, and SrHfS_3_, and any ternary phases of the Sr–Ti–S, Ca–Zr–S, and Ca–Hf–S systems *via* colloidal methods proved unsuccessful, previous work does show that solution-based nanocrystal syntheses offer the unique ability to stabilize materials in metastable phases by taking advantage of non-equilibrium reaction kinetics and the influence of surface energies on the system thermodynamics. Additionally, cation-exchange methods are commonly used to access metastable polymorphs of semiconductor nanocrystals.^51,53^ Thus, given the right combination of precursors, precursor addition order and rate, ligands, and reaction parameters like time, temperature, and heating rate (among others), it would seem that the colloidal nanocrystal formation of these species could become possible.

### Film fabrication and optoelectronic properties of ABS_3_ compounds

**Fig. 5:**
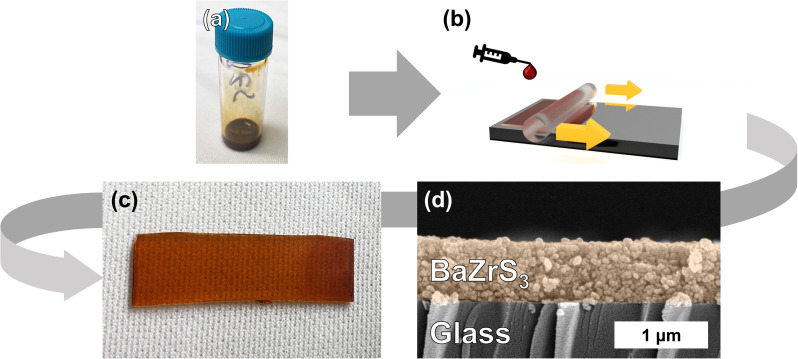
Blade coating procedure to produce BaZrS_3_ nanoparticle films. (a) Shows a typical ink of suspended BaZrS_3_ nanoparticles, (b) illustrates the blade coating procedure, (c) shows a photograph of the coated film on a glass substrate, and (d) shows a cross-section SEM image of the same film.

To investigate the functional properties of these compounds, methods to deposit this material as a thin film are highly desirable. By taking advantage of one of the key properties of colloidal nanomaterials, we are able to achieve this. Under the proper reaction conditions, these ABS_3_ compounds can possess high levels of colloidal stability (remaining visually colloidally stable for greater than 1 year for the case of BZS and BHS). Upon favorable synthesis of SP-BZS (in terms of colloidal stability), we then dried the NPs and resuspended in an appropriate amount of 1-hexanethiol to obtain a BZS NP ink at a concentration of 100 mg mL^−1^. Shown in Fig. 5 is the result of coating the film on a piece of Corning Eagle XG glass. To the best of our knowledge, this film is the first fully continuous, fully solution-processed film of BaZrS_3_. Films of BaZrS_3_ from colloidal routes have been reported before, but a solid-state synthesis step was required to first synthesize the chalcogenide perovskite.^54^ The added advantage of producing these films *via* solution-processed methods allows these films to be produced at comparatively low temperatures.

**Fig. 6:**
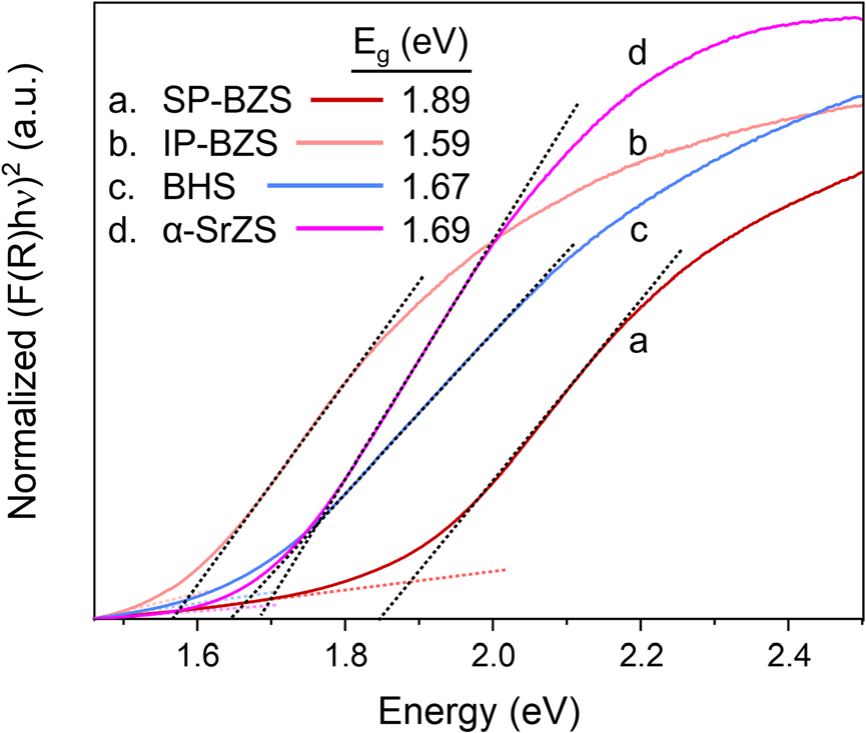
Using the Kubelka–Munk method for diffuse reflectance measurements, the band gap for each of the indicated species is determined. The band gap for each was determined from the intersection of the straight-line regions above and below the band gap to account for the material's absorption and measurement background, respectively.

To measure the absorption properties of each of the synthesized compounds, UV-vis spectroscopy was performed and results are shown in Fig. 6, S11 and S12.† Using the Kubelka–Munk method for diffuse reflectance measurements, we can see that the SP-BZS NPs possess a bandgap of 1.89 eV that falls within the expected range of ∼1.75–1.95 eV for orthorhombic distorted perovskite *Pnma* structure of BaZrS_3_,^14^ indicating the synthesis of high-quality BaZrS_3_ perovskite nanoparticles. On the other hand, IP-BZS is found to have a lower bandgap of 1.59 eV signifying that the IP-BZS is distinct from the orthorhombic phase of BaZrS_3_. Similarly, the BHS nanoparticles have a much lower bandgap of 1.67 eV as compared to about 2.1 eV for the bulk distorted perovskite phase of BaHfS_3_, indicating the BHS phase synthesized here is also distinct from the orthorhombic phase of BaHfS_3_.^14^

The measured bandgap of 1.69 eV for the α-SrZrS_3_ nanocrystals is found to be higher than previously reported values of 1.52–1.53 eV.^55^ This may be due to quantum confinement effects which may be present due to the small diameter of the SrZS needles (∼5–10 nm). To support this argument, we calculated the de Broglie wavelength for electrons in α-SrZrS_3_ using the equation, 
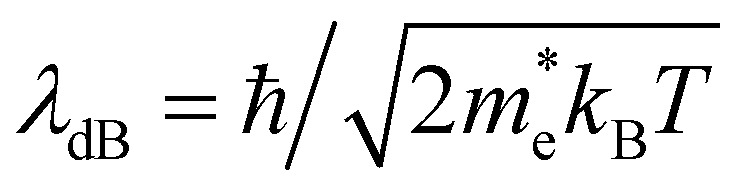
,^56^ where *ℏ* is the reduced Planck constant (*ℏ* = *h*/2π), 
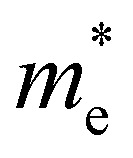
 is the effective electron mass, *k*_B_ is the Boltzmann constant, and *T* is temperature. If any dimension of a nanomaterial approaches that of the de Broglie wavelength (or exciton Bohr radius), size-dependent properties caused by quantum confinement begin to take effect. Using data from Kumar *et al.* for effective electron mass,^57^ we find *λ*_dB_ = 2.14 nm, comparable to the needle diameters that we see *via* TEM imaging. Quantum confinement effects are also commonly observed in Si nanowires as their diameter decreases showing changes in band gap and band structure changing the Si nanowires from an indirect (as observed for bulk Si) to a direct band gap material below a certain wire diameter.^56^ While currently unclear what changes may be occurring to the band structure of α-SrZrS_3_ at these small needle diameters, we have also measured the bandgap using the Kubelka–Munk method in Fig. S11† for α-SrZrS_3_ as if it were an indirect band gap material. A slight decrease in its band gap as compared to the analysis in Fig. 6 is determined, although still above that previously reported for bulk α-SrZrS_3_.^55^ We also include the measurements for IP-BZS and BHS in Fig. S11† since it is currently not clear whether these irregular phase materials have a direct or indirect band gap. Like α-SrZrS_3_, a slight decrease in the band gap is also interpreted from these analyses. Additionally, we include the full range of the raw data collected during UV-vis measurements in Fig. S12.†

Studies into the photoluminescent (PL) properties of these colloidal materials became difficult due to the luminescent nature of the organosulfur byproduct(s) formed during the nanoparticle syntheses from the reaction between OLA and CS_2_. As shown in Fig. S14,† the product(s) of the reaction between OLA and CS_2_ at 300 °C gave a broad PL spectrum with a peak centered near 2.2 eV. IP-BZS particles with poor washing after the reaction also show an intense PL spectrum with a peak in the neighborhood of 2.2 eV (Fig. S15b†). However, with increasing washings, the intensity of the PL spectrum decreased along with the peak location moving towards a lower value of 2 eV. Interestingly, both the IP-BZS and α-SrZrS_3_ nanoparticles prior to thorough washing showed nearly identical PL spectrum with a peak location near 2 eV (Fig. S13†) while the measured bandgap of each of these materials is much lower than 2 eV (Fig. 6). As such, we believe the PL spectrum obtained from the synthesized nanoparticles is likely a result of the residual, luminescent organosulfur byproduct remaining on the surface of the synthesized nanoparticles. It is worth noting that Yang *et al.* also observed PL from their BaZrS_3_ nanoparticles with a peak position of 2.08 eV.^32^ A careful examination and further study of the PL spectrum from the BaZrS_3_ nanoparticles synthesized by the currently known methods, including ours, is needed. Further experimental details are given in the ESI section ‘Supplementary discussion on photoluminescence’ in the context of Fig. S13–S15 and Table S3.†

In fabricating thin films of BaZrS_3_, we also investigated the potential for sintering and grain coarsening of the nanoparticle films *via* ampule sulfurization experiments. For these experiments, we followed a methodology similar to some of our previous works,^30,36,58^ using excess elemental sulfur in combination with an oxygen trap in HfH_2_. The use of these two species creates an atmosphere in the ampule that possesses a complex identity—from varying S_*n*_ chain lengths^59^ and H_2_S resulting from the reaction of elemental sulfur with HfH_2_—not to mention any H_2_O that may form from residual oxygen and oxygen-containing species in the ampule.^30^ During these experiments, we observed differing results depending on the properties (*i.e.* thickness and Ba/Zr ratio, for example) of the coated film; however, the most consistent result was severe island formation of BaZrS_3_ grains. We also observed *via* pXRD that IP-BZS NPs undergo a transformation into higher-crystallinity SP-BZS grains during sulfurization. The results of these experiments are shown in Fig. S16 and S17† where pXRD and SEM data of the sulfurized films are shown. The island growth and non-continuous films are similar to what we have observed from our previous works using solution-based methods. As stated before, sulfurization of films in this manner is performed in an environment with a chemically complex atmosphere which can make understandings of the various mechanisms involved, and thus optimizations, difficult. Regardless, this leaves room for further study of not only this sulfurization mechanism, but of other methods (*i.e.* flow systems, use of different sulfur species like H_2_S or CS_2_, *etc.*) to obtain higher quality sintered films of BaZrS_3_ and other chalcogenide perovskites—important for fabrication of a functional device in which subsequent layers need to be deposited on top of the perovskite layer, should a continuous, sintered film be desired.

## Conclusions

In this work, we have developed a generalized protocol for synthesizing various ABS_3_ species, including BaZrS_3_, BaTiS_3_, SrZrS_3_, and SrHfS_3_ as well as colloidal materials from the Ba–Hf–S system—materials that consist of highly oxophilic elements, especially the group IV transition metals Ti, Zr, and Hf. To the best of our knowledge, we have provided the first comprehensive study on the colloidal synthesis of a handful of these AE–IVB–S materials—with solution-processing of these materials described as a highly unlikely feat only just a couple of years ago. We were also able to unravel the phenomenon regarding BaZrS_3_ phase formation, notably giving us control to form the standard phase over the irregular phase by taking advantage of the reaction system kinetics. With high-quality BaZrS_3_ perovskite nanoparticles, we can ensure future studies are being conducted with the proper material—an important step as we have shown, at the very least, different absorption properties between the two BZS phases—opening the door for exciting studies, such as how nanoparticle surface ligands may be dictating the luminescent properties of these materials. In addition to the nanoparticle syntheses, we have demonstrated the colloidal stability of the synthesized BaZrS_3_ nanoparticles by depositing the nanoparticles as a uniform thin film. Finally, we showed that these nanoparticles can also undergo sintering and growth during furnace sulfurizations. While this can be used to access the orthorhombic distorted perovskite phase of BaZrS_3_ from its irregular phase, the poor film morphology obtained from the sintered films, leaves room for further study of sintered, polycrystalline films of BaZrS_3_. We believe that the work in this study will further contribute and bring the chalcogenide perovskite community ever closer to optoelectronic device fabrication for the realization of truly earth-abundant functional materials.

## Data availability

The data supporting this article have been included as part of the ESI† which includes the experimental methodology, additional information on the purification and handling of certain precursor materials, TEM/STEM images, reaction data including pXRD and Raman data, additional UV-vis data, a supplementary discussion on studies into the photoluminescence of the materials synthesized in this study, and some additional pXRD, Raman, and SEM data of samples annealed in a sulfur atmosphere.

## Author contributions

DCH: conceptualization, data curation, formal analysis, investigation, methodology, visualization, writing – original draft, writing – review & editing. SA: data curation, investigation, validation, writing – review & editing. KCV: data curation, writing – review & editing. IMA: data curation, validation, investigation. AAP: data curation. MCU: investigation. SCB: conceptualization, writing – review & editing. RA: conceptualization, funding acquisition, supervision, writing – review & editing.

## Conflicts of interest

There are no conflicts to declare.

## Supplementary Material

SC-OLF-D4SC06116K-s001
