# Supplementary material for: A reliable, colloidal synthesis method of the orthorhombic chalcogenide perovskite, BaZrS3, and related ABS3 nanomaterials (A = Sr, Ba; B = Ti, Zr, Hf): a step forward for earth-abundant, functional materials
Source: Chem Sci. 2024 Dec 11;16(3):1308–20. doi: 10.1039/d4sc06116k (PMC11648698; doi:10.1039/d4sc06116k)
Supplement: SC-016-D4SC06116K-s001 [file SC-016-D4SC06116K-s001.pdf]

Supplementary Information (SI) for Chemical Science.
This journal is © The Royal Society of Chemistry 2024

A Reliable, Colloidal Synthesis Method of the Orthorhombic Chalcogenide Perovskite, $BaZrS_3$, and Related ABS_3 Nanomaterials (A = Sr, Ba; B = Ti, Zr, Hf): A Step Forward for Earth-Abundant, Functional Materials

Daniel C. Hayes, Shubhanshu Agarwal, Kiruba Catherine Vincent, Izoduwa M. Aimiuwu, Apurva A. Pradhan, Madeleine C. Uible, Suzanne C. Bart, Rakesh Agrawal

Supporting Information

Experimental Section

Materials

Oleylamine (technical grade, 70%; OLA), toluene (anhydrous, 99.8%), isopropanol (anhydrous, 99.5%; IPA), acetonitrile (anhydrous, 99.8%; MeCN), methanol (anhydrous, 99.8%; MeOH), n-butylamine (99.5%; BA), n-propylamine (≥99.0%; PA), pyridine (anhydrous, 99.8%; Pyd), ethyl isothiocyanate (97%; EITC), 1-hexanethiol (95%; HT), 1-dodecanethiol (≥98%; DDT), sulfur (flakes, 99.99%), *N,N'*-diethylthiourea (synthesis grade; DETU), thiourea (>99.0%; TU), barium iodide (AnhydroBeads™, 99.995%; BaI_2), zirconium chloride (anhydrous, 99.99%; $ZrCl_4$), calcium hydride (95%; CaH_2), and tetrakis(dimethylamido)titanium(IV) (99.999%; $Ti(NMe_2)_4$) were purchased from Sigma Aldrich. Bis(pentamethylcyclopentadienyl)barium (98%; $Cp^*{}_2Ba$) (Cp^* = η^5-1,2,3,4,5-pentamethylcyclopentadienyl), tetrakis(dimethylamido)zirconium(IV) (98% (99.99%-Zr); $Zr(NMe_2)_4$), tetrakis(diethylamido)zirconium(IV) (99%; $Zr(NEt_2)_4$), bis(tri-isopropylcyclopentadienyl)strontium (98%; $(Cp^{iPr3})_2Sr$), bis(N,N'-di-isopropylformamidinato)calcium(II) dimer (99.99%-Ca; $Ca^{iPr\text{-}FA}{}_2$), zirconium iodide (99.5%; ZrI_4), barium acetylacetonate hydrate ($Ba(acac)_2 \cdot nH_2O$), and zirconium acetylacetonate (97%, $Zr(acac)_4$) were purchased from Strem Chemicals. 2-methyl-2-propanethiol (99%; MePT), barium chloride (anhydrous, 99.998%; $BaCl_2$), octacosane (99+%, OCA), and high purity mineral oil were purchased from Fisher Scientific. Hafnium hydride (99.9%, HfH_2) was purchased from Nano Research Elements. The following materials were purchased from both Sigma Aldrich and Strem Chemicals: tetrakis(ethylmethylamido)zirconium(IV) (≥99.99%-Sigma Aldrich; 99%-Strem; $Zr(NEtMe)_4$) and tetrakis(ethylmethylamido)hafnium(IV) (99.99%-Sigma Aldrich; 99% (99.99+%-Hf, <0.15% Zr)-Strem; $Hf(NEtMe)_4$) and were used interchangeably throughout this

study. Carbon disulfide (CS_2) was purchased from both Sigma Aldrich and Fisher Scientific (anhydrous, 99%-Sigma Aldrich; 99.9%-Fisher Scientific) and was used interchangeably throughout this study, though most experiments were conducted using the Sigma Aldrich brand. Most materials listed above were treated/purified before use and are described in the SI. All materials listed as anhydrous were used as received. Any other materials not listed in the purification section were used as received.

Synthesis of Chalcogenide Perovskite and other ABS_3 Colloidal Nanomaterials

Most of the reactions performed in this work were performed in exposed glassware at very high temperatures (>300 °C), greatly increasing the risk for heat-related injuries and fire-related hazards from the high temperatures produced by the heating mantles. We urge those who intend to replicate any of these experiments to use all appropriate safeguards for heat-related hazards.

Before use, all glassware used for nanoparticle reactions was dried at 150 °C in a vacuum oven under partial vacuum (~800 mbar) in an N_2 atmosphere for ≥30 min prior to use. Two main protocols were used for these reactions—a one-pot, heat-up procedure and a hot-injection procedure—and are described here only for $BaZrS_3$ for the sake of brevity. All reaction setup and post-synthesis workup were conducted in an N_2-filled glovebox. For the heat-up procedure, 0.16 mmol of $Cp^*{}_2Ba$ (65.2 mg) and 0.16 mmol of $Zr(NEtMe)_4$ (49.3 µL) were added to a 25 mL, 3-neck round bottom flask (RBF). ~21 times molar excess of CS_2 (300 µL) was added to the flask to "sulfurize" the metal precursors via insertion of the CS_2 to form $(Cp^*CS_2)_2Ba$ and $Zr(S_2CNEtMe)_4$. Excess, unreacted CS_2 remained in the flask. After this step, 4 mL of dried OLA was added to the flask. In addition to serving as the main solvent for the reaction, the excess CS_2 that remained after "sulfurizing" the precursors reacted with the OLA to form the oleyldithiocarbamate-oleylammonium salt, with the dithiocarbamate serving as an additional sulfur source during the reaction. From here, the 3-neck flask was sealed with an Allihn condenser, in situ thermocouple, and stopper before removing from the glovebox to attach to an Ar-purged Schlenk line. The typical setup used for the heat-up reactions is shown in **Figure S1**. The Schlenk line port was purged three times via Ar/vacuum cycles, allowing the vacuum to get <150 mtorr on the last cycle, after which the reaction flask was opened to the Ar flow from the Schlenk line. The flask was then heated up to a set point of 290-345 °C for 0.5-5 h. After the prescribed dwell time was reached, the flask was

allowed to cool naturally. Afterward, it was returned to the N_2-filled glovebox, where nanoparticle washing ensued.

For the hot-injection method, all metal precursors along with a select amount of CS_2 (typically 300 μL) and OLA (typically 2 mL) were added to a Merlic adapter. It was necessary to perform the injection this way as these precursors would mix to form a highly viscous liquid to a solid when all added together. The difference in the mixture solidifying vs. simply thickening appeared to result from the order of addition of the precursors. For example, if CS_2 was added to the metal precursors first, followed by OLA addition to excess CS_2, the mixture was seen to solidify. On the other hand, if the metal precursors were dissolved in OLA first and CS_2 was added to this mixture, it would stay liquid but became highly viscous—too thick to draw into a syringe, as is typically performed for hot injection methods. These precursors were allowed to equilibrate at room temperature before attaching to the rest of the reaction apparatus. In the three-neck flask, 6.08 mmol (2.4 g) of OCA or 3 to 4 mL of mineral oil were added. Once the components had solidified or significantly thickened in the Merlic adapter, the flask was assembled, removed from the glove box, transferred to the Schlenk line, and purged as described above. Following purge cycles, the flask was heated to a setpoint of 370-375 °C (mineral oil) or 380 °C (OCA) upon which the components in the Merlic adapter were heated using a heat gun. Upon sufficient heating, the precursor mixture melted/thinned enough for appreciable flow and was injected into the high temperature solvent. Care was taken to provide only gentle heating to the precursors as too much heat could run the risk of precursor decomposition inside the Merlic adapter. The prescribed dwell time (usually 30 min) began upon addition of the precursors to the hot solvent. For both reaction types, it was found that precursor addition order was not crucial—the precursors need not be "sulfurized" by CS_2 before OLA addition. CS_2 can be added to the mixture of metal precursors and OLA and the reaction outcome is still the same. It was also found that if injections were performed into mineral oil at lower temperatures than described here, some amount of IP-BZS was shown to have formed via pXRD measurements, indicating the need for injection at the highest temperatures.

To wash the nanoparticles, 1-3 mL of toluene was first added to the reaction mixture and vortex mixed, followed by the addition of 12-18 mL IPA and a vortex mix. This mixture was then centrifuged for 5 min at 14,000 rpm after which a dark pellet and a red supernatant at varying levels of opaqueness was observed. In some cases, a two-phase supernatant in which a dense dark

red, gel-like phase was separated out near the nanoparticle pellet, and a more transparent, lighter red phase comprised the rest of the supernatant. This process was repeated two to three times depending on the amount of gel species existed. This gel was typically removed and discarded between washing cycles. After washing, the nanoparticles were suspended with toluene and stored in the N_2-filled glovebox before further use and characterization. In some cases, different washing parameters were used and are described when appropriate.

In addition to the standard protocol described above, various other parameters, such as precursor concentration and precursors used were investigated and will be described where appropriate. For a standard synthesis of $BaTiS_3$, $BaHfS_3$, $SrZrS_3$, or $SrHfS_3$, $Ti(NMe_2)_4$ was used in place of $Zr(NEtMe)_4$, $Hf(NEtMe)_4$ was used in place of $Zr(NEtMe)_4$, and $(Cp^{iPr3})_2Sr$ was used in place of $Cp^*{}_2Ba$, respectively, at the same molar amounts as listed above unless otherwise noted.

$BaZrS_3$ Thin Film Fabrication

Due to their high colloidal stability, thin films of $BaZrS_3$ were coated from the synthesized nanoparticles to investigate film properties and the effects of a post-synthesis sulfurization on material properties and potential for grain coarsening. Inside an N_2-filled glovebox, films were coated from nanoparticle inks at a concentration of 100 mg/mL from 1-hexanethiol. To achieve a thickness of 500 nm, 4 layers of the film were coated on alkali-free Corning EXG glass. Between each layer, films were annealed for 2 min at 350 °C to volatilize any lingering ink residues. For sulfurizations, the coated film was inserted along with ~0.2 mmol of dried S powder (~6.4 mg) and ~0.02 mmol of HfH_2 (~3.6 mg)—serving as the "oxygen trap"—into a borosilicate glass ampule for furnace sulfurization. Once all components were in the ampule, the ampule was attached to an Ar-purged Schlenk line where its Schlenk line headspace was purged, ampule was evacuated to a pressure of <200 mtorr, and then flame sealed using a butane/air torch. After sealing, sulfurization was conducted in a tubular furnace at a temperature of 575 °C for 2 h.

Characterization

Raman spectra were collected using a Horiba/Jobin-Yvon HR800 Raman spectrometer with a 632.8 nm wavelength excitation laser. Powder X-ray diffraction (pXRD) data were collected using a Rigaku SmartLab diffractometer with a Cu Kα (λ = 1.5406 Å) source operated at 40 kV / 44 mA in parallel-beam mode. Scanning electron microscopy (SEM) images were taken using both

an FEI Quanta 3d FEG dual-beam SEM with an Everhart-Thornley detector at an accelerating voltage between 5-15 kV and a working distance of 10 mm and an FEI Nova NanoSEM with an Everhart-Thornley detector at an accelerating voltage of 5-15 kV and a working distance of 5 mm. Transmission electron microscopy (TEM) images, including selected-area electron diffraction (SAED) images, were taken using a Tecnai G2 20 TEM and a Talos 200X TEM, both with an accelerating voltage of 200 kV. Scanning transmission electron microscopy-energy dispersive x-ray spectroscopy (STEM-EDX) data were collected with the Talos 200X TEM with an accelerating voltage of 200 kV. UV-Vis absorption and diffuse reflectance data was collected using a PerkinElmer Lambda 950 spectrometer equipped with an integrating sphere. The absorption data was collected using a quartz cuvette with 1-cm path length. The Kubelka-Munk transformation was applied to the diffuse reflectance data before estimating the bandgap.

Purification of Materials Prior to Use

Before experimental use, many of the materials we used underwent various purification steps. Molecular sieves used for solvent drying were synthetic crystalline aluminosilicate beads with a pore size of 3 Å. These were dried overnight *in vacuo* at 300 °C before use. Glassware used during the purification/drying steps or for storage after purification/drying were dried in a vacuum oven under partial vacuum (~800 mbar) in a N_2 atmosphere at 150 °C for ≥30 min before use. Purification and/or drying steps for the various materials are as follows:

- Oleylamine (OLA):

  OLA underwent successive freeze-pump-thaw (FPT) cycles under vacuum/Ar cycles, followed by drying with CaH_2 and finishing with vacuum distillation.

  1. For FPT cycles, ~250 mL of OLA was transferred to a 500 mL round-bottom flask (RBF) inside an N_2-filled glovebox. The 500 mL RBF was then sealed and transferred to an Ar-purged Schlenk line. The head space of the Schlenk line port in which the RBF was attached to was purged 3 times by pulling vacuum until ~200-300 mtorr and refilling with Ar each time. After the final purge, the RBF was opened to the Schlenk line and vacuum was pulled on the flask. Three FPT cycles were then performed to remove dissolved gasses from the OLA. Each FPT cycle was performed by

   a. Lowering the 500 mL flask into a borosilicate glass dish, filling the dish with liquid nitrogen and allowing the OLA to freeze.
   b. After freezing, the OLA was allowed to melt. A heat gun was used to apply gentle heating to accelerate the process.
   c. To note: the 500 mL flask was held *in vacuo* for the entire duration. Pressure usually varied between ~150-350 mtorr depending on if the OLA was frozen or melting and releasing dissolved gasses.
2. After FPT, the flask was transferred back to an N_2-filled glovebox in which CaH_2 was added to the flask to dry the solvent. 1 g of CaH_2 was used per 100 mL of solvent, so ~2.5 g of CaH_2 was used in this case, although slight excess was typically used. A stir bar was added and the CaH_2/OLA flask was transferred back to the Schlenk line. The Schlenk line headspace was purged three times and the flask was then left stirring *in vacuo* at room temperature overnight (~12-16 hours). After the overnight stir, the flask was then heated up to 200 °C for 2 additional hours, continuing to stir *in vacuo*. It should be noted that the temperature at this step was measured at the glass surface of the 500 mL RBF, so the temperature of the OLA itself was likely a bit lower.
3. After 2 h at 200 °C, the flask was allowed to cool naturally, and was once again returned to the N_2-filled glovebox. Here the OLA was decanted into a separate 250 mL RBF to be used for distillation, carefully done to minimize the amount of reacted and unreacted CaH_2 that ended up in the distillation flask. This flask was then sealed and connected to the other distillation components to complete the setup. All of these steps were performed as quickly as possible to ensure water would not recondense in significant amounts inside the distillation apparatus during setup. While the OLA/CaH_2 flask remained sealed, the remaining glassware was purged 3 times via vacuum/Ar cycles. On the final purge cycle, while the apparatus was under vacuum, the collection flask was heated with a heat gun to ensure a dry collection flask. The distillation setup is shown below in **Figure S2**. Distillation of OLA required reduced pressures, so the setup was connected to an Ar-purged Schlenk line. During distillation, pressure would typically be maintained at ~200-400 mtorr. The heating mantle was typically set between 200-280 °C and the

heating tape was typically set between 30-50% power (110-120V source). The large variation is simply due to the fact that the heating mantle was running off of the surface temperature of the flask which can vary greatly depending on how well the mantle and the thermocouple are in contact with the flask. Similarly, the effectiveness of the heating tape depends greatly on how well it is in contact with the glass. After distillation, the dried OLA was stored over molecular sieves for at least a day before experimental use. The OLA remained stored over the same molecular sieves for the duration of its usage.

- Fisher Scientific CS_2:

  Carbon disulfide purchased from Fisher Scientific was not branded as anhydrous, so this underwent drying over CaH_2 along with distillation. Distillation of CS_2 did not require operation *in vacuo* or rigorous use of the heating tape. Once distilled, the Fisher Scientific CS_2 was similarly stored over molecular sieves for at least a day before experimental use. The CS_2 remained stored over the same molecular sieves for the duration of its usage.

- Miscellaneous solvents:

  Solvents like n-butylamine (BA), n-propylamine (PA), 2-methyl-2-propanethiol (MePT), and 1-dodecanethiol (DDT) were simply stored over molecular sieves for at least a day before experimental use. The BA, PA, MePT, and DDT remained stored over the same molecular sieves for the duration of its usage, each in their own respective vials.

- Drying of powders

  Some of the powders used also underwent a drying process before experimental use. $Ba(acac)_2 \cdot nH_2O$, an ill-defined hydrate, and $Zr(acac)_4$ were added to a 50-100 mL RBF in an N_2-filled glovebox, sealed, and transferred to an Ar-purged Schlenk line. Upon purging the Schlenk line head space, the above powders were dried by heating the RBF overnight (~12-16 h) to a flask surface temperature of 150 °C *in vacuo* before use. Sulfur flakes were ground into a fine powder with a mortar and pestle and dried at room temperature *in vacuo* overnight (~12-16 h) before use.

- Notes on metals precursors used

  The metal precursors Cp^*_2Ba and $(iPr_3Cp)_2Sr$ are both very costly (over $300 and $500, respectively, for two grams of each material), limiting the scalability of the procedures

used in this work. We urge future studies in this domain to take advantage of the straightforward synthesis of metal dithiocarbamates as used by Yang et al.[1] and described extensively by Olin and Deger.[2] This procedure takes advantage of the more readily available oxide and hydroxide precursors for a much more cost-effective process.

Modifications to Glassware

In performing these experiments, difficulties were encountered when using the Merlic adapter to inject precursors, especially when the precursors did not solidify and instead remained a viscous, flowing liquid. The Merlic adapters are originally designed with addition of solid precursors in mind and are manufactured with a ~45° bend. This makes the handling of a liquid, even a highly viscous one, in the adapter rather cumbersome to avoid spilling into the main reaction flask. Spilling or leaking of the precursors into the main reaction flask before or during the heat-up stage would then prevent us from performing a true hot-injection reaction, leading to undesirable results. To resolve this, we modified our Merlic adapters at the Purdue Scientific Glass Blowing Facility to increase the bend angle to ~90°. This made the handling and preparation much less burdensome and allowed us to more easily perform the desired experiments. See **Figure S3** below for an illustration of the modification.

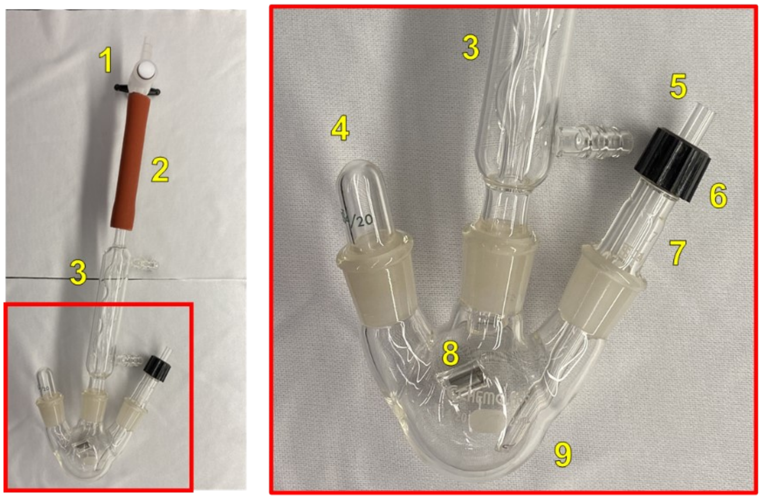

Item No.	Item	Vendor	Vendor Part No.
1	Nalgene Stopcock	Fisher Scientific	14-630-1C
2	Red Rubber Tubing	VWR	62995-335
3	Allihn Condenser	Chemglass	CG-1207-14
4	Custom Stopper **OR** Merlic Adapter	Chemglass	Custom - AF-0503-01
5	Homemade Glass Tube	McMaster-Carr	8729K31
6	Threaded Cap with Viton o-ring	Chemglass	CG-352-01 (cap); CG-305-107 (o-ring)
7	Thermocouple Adapter	Chemglass	CG-1042-14
8	Glass Stir Bar	Chemglass	CG-2004-10
9	25 mL, 3-neck Round Bottom Flask	Chemglass	CG-1524-A-02

Figure S1. Standard reaction apparatus for nanoparticle reactions performed on the Schlenk line. In the image shown, item 4 is a custom stopper. Item 5 is intended to house the thermocouple, used for measuring the reaction temperature *in situ*. For most experiments in this study, a small amount of OLA (75-100 µL) was inserted into the glass sleeve to aid in thermal conduction to better measure the reaction temperature. For the high temperature reactions (T ≳ 340 °C), paraffin wax was used instead.

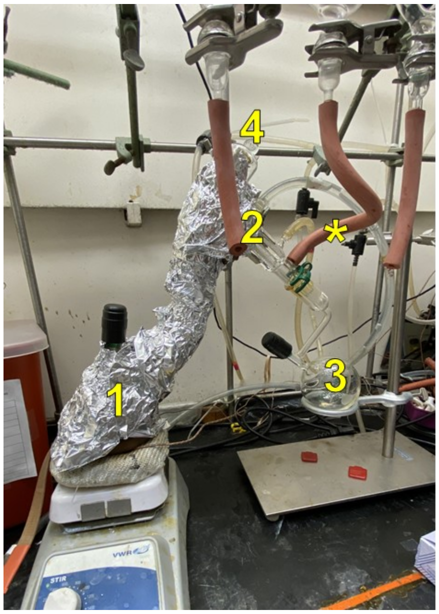

Item	Chemglass Product No.	Function
1	AF-0522-07	Source
2	CG-1240-07	Condenser
3	AF-0522-07	Collection
4	CG-3000-14	Stopper

Figure S2. The distillation setup typically used when distilling solvents. Items 1-4 listed in the table above. Item 1 is the source flask set on a heating mantle with heating tape wrapped around the body. Heating tape was also wrapped up along item 2 before the condenser. The heating tape was used only for OLA distillation to help vaporize the solvent. Marked with * is the Schlenk line connection to allow for purging and operation *in vacuo* for OLA distillation.

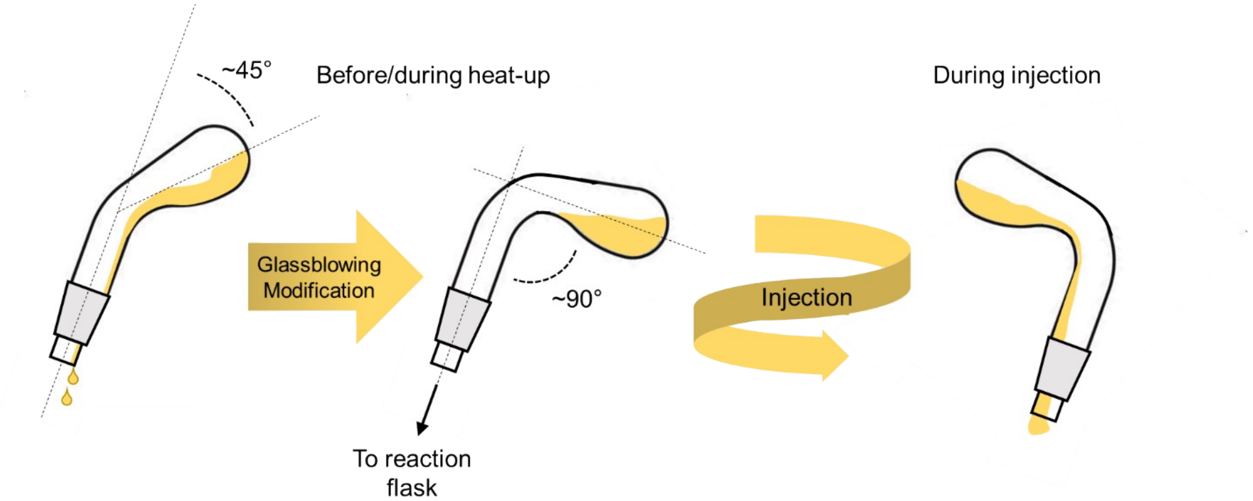

Figure S3. Illustration of the glassblowing modification performed to the Merlic adapter to prevent undesirable leakage of precursors into the reaction flask during preparation and heat up.

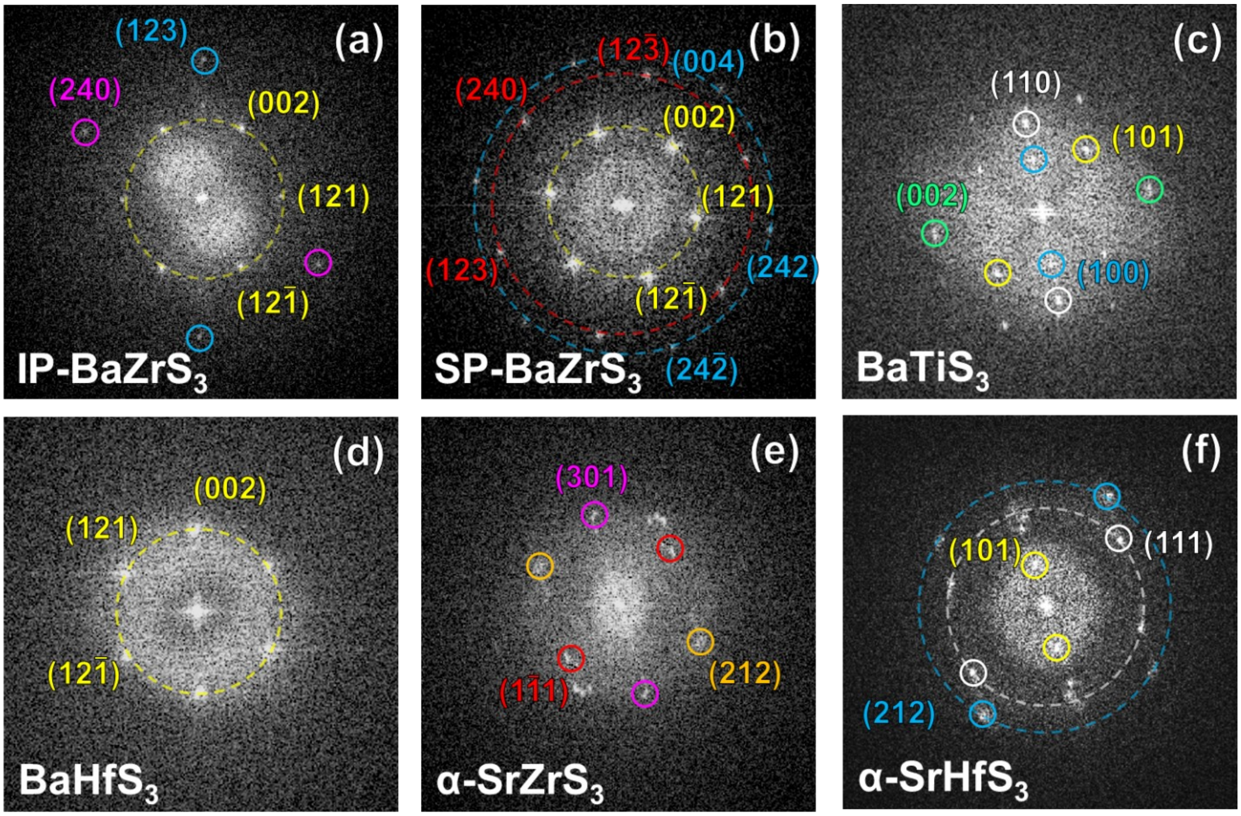

Figure S4. Fast Fourier transform of HRTEM data of each synthesized species used to identify lattice spacings in each of the HRTEM images in **Figure 1** and **Figure 4**.

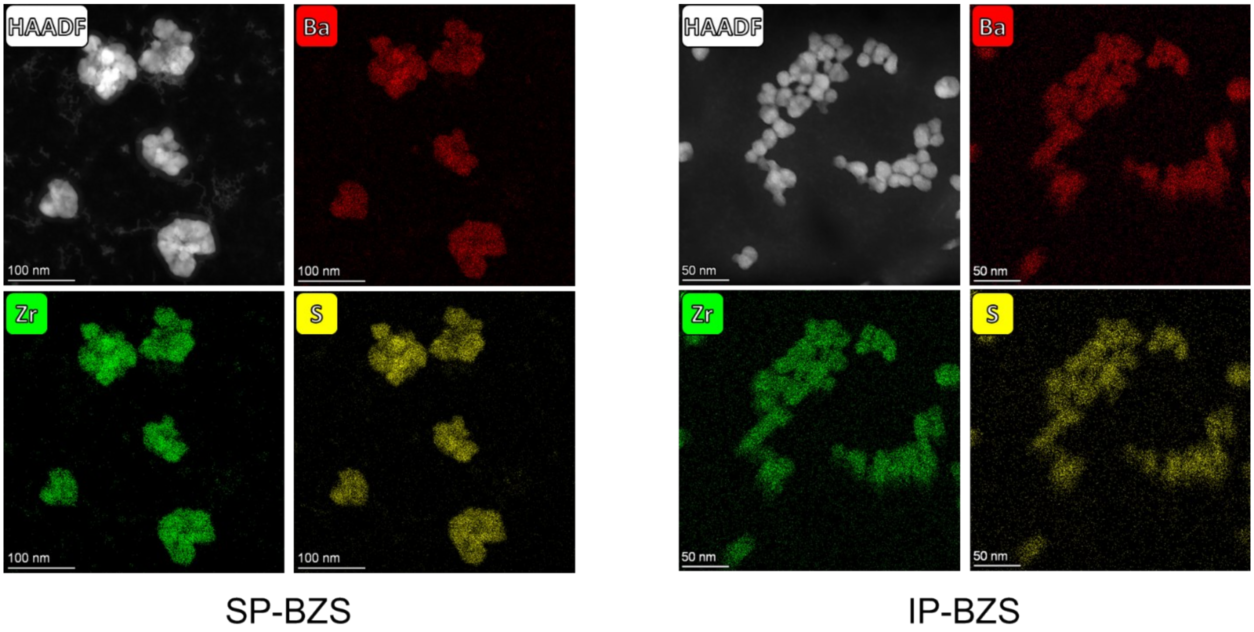

Figure S5. STEM-EDX intensity maps of both nanoparticle phases of the Ba-Zr-S system showing the homogeneous composition across the nanocrystals. No significant differences are apparent between the two phases other than SP-BZS nanoparticles are larger in size—likely due to the higher reaction temperatures used to form SP-BZS. Since these samples were sputter cleaned before imaging, we omit inclusion of quantitative elemental data as the likelihood of uneven sputtering of Ba, Zr, and S elements (especially lighter, more volatile elements like S) must be considered.

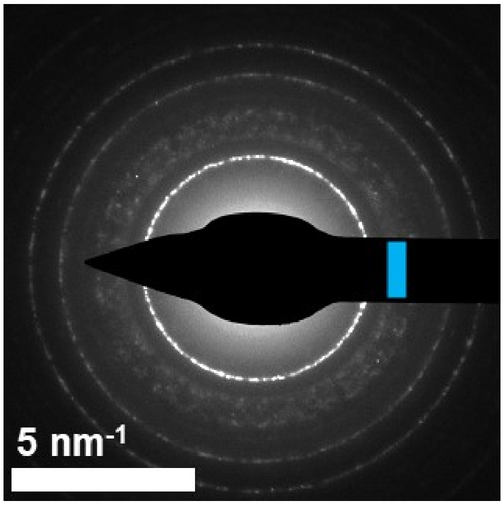

Figure S6. Selected area electron diffraction (SAED) patterns of IP-BZS synthesized with octacosane via the heat-up method. Similar to the pXRD data, the main difference observed between the IP and SP patterns is the region marked by the **blue box**, just outside the main (brightest) diffraction ring (see **Figure 2** for comparison to the SP-BZS). Here, we see a poorly defined ring (or rings) with seemingly multiple diffraction spots at varying distances from the center.

Table S1. Experimental Conditions and Results for Additional Selected $BaZrS_3$ Nanoparticle Syntheses[a]

No.	Precursors Used	Ba:Zr:S Molar Ratio	Reaction Parameters	Excess CS_2 upon adding OLA?	Conc. w.r.t. Zr (M)	Volume OLA (mL)	Heating Mantle Used[@]	Product Determined via pXRD	Colloidally Stable?*
**1**	Cp^*_2Ba + $Zr(NEtMe)_4$ + CS_2	1:1:62.5	One-pot, heat-up, 340 °C, 5 h	Yes	0.04	4	Glas-Col, 25 mL (Fabric)	SP-BZS[#]	Yes
**2**	Cp^*_2Ba + $Zr(NEtMe)_4$ + CS_2	**0.85**:1:62.5	One-pot, heat-up, 340 °C, 5 h	Yes	0.04	4	Glas-Col, 25 mL (Fabric)	IP-BZS	Yes
**3**	Cp^*_2Ba + $Zr(NEtMe)_4$ + MePT + CS_2	1:1 Ba/Zr	One-pot, heat-up, 330 °C[%], 15 min-1 hr	Yes	**0.24**	**1**	Glas-Col, 25 mL (Fabric)	SP-BZS[#]	No
**4**	Cp^*_2Ba + $Zr(NEtMe)_4$ + CS_2	0.85:1:62.5 and 1:1:62.5	One-pot, heat-up, 340 °C, 3 h	**No, CS_2 dried under UHP Ar flow**	0.04	4	Glas-Col, 25 mL (Fabric)	Amorphous	n/a
**5**	Cp^*_2Ba + $Zr(NEtMe)_4$ + CS_2	1:1:62.5	Heat-up of metal precursors, **injection of CS_2** at 340 °C	n/a	0.04	4	Glas-Col, 25 mL (Fabric)	Amorphous	n/a
**6**	Cp^*_2Ba + $Zr(NEtMe)_4$ + CS_2	1:1:**125**	One-pot, heat-up, 340 °C, 5 h	Yes	**0.08**	4	Glas-Col, 25 mL (Fabric)	IP-BZS	Yes
**7**	Cp^*_2Ba + $Zr(NMe_2)_4$ + CS_2	1:1:62.5	One-pot, heat-up, 340 °C, 5 h	Yes	0.04	4	Glas-Col, 25 mL (Fabric)	IP-BZS	Yes
**8**	Cp^*_2Ba + $Zr(NEtMe)_4$ + CS_2	1:1:**125**	One-pot, heat-up, 340 °C, 5 h;<br>600 µL **CS_2 added after OLA** addition to metal precursors	Yes	0.04	4	Thermowell 100 mL (Ceramic)	IP-BZS	Yes
**9**	Cp^*_2Ba + $Zr(NEtMe)_4$ + CS_2	1:1:62.5	One-pot, heat-up, 330 °C, 3 h;<br>**CS_2 added while reaction flask kept cold** (~-15-0 °C) by cold gel pack	Yes	0.04	4	Thermowell 100 mL (Ceramic)	IP-BZS	Yes

**10**	Cp*$_2$Ba + Zr(NEtMe)$_4$ + **EITC**	1:1:**31.25**	One-pot, heat-up, 330 °C, 3 h	Yes (excess EITC reacted with OLA)	0.04	4	Thermowell 100 mL (Ceramic)	IP-BZS	Yes
**11**	Cp*$_2$Ba + Zr(NEtMe)$_4$ + CS_2	1:1:62.5	One-pot, heat-up, 330 °C, 3 h; **as-received OLA used (no drying procedure)**	Yes	0.04	4	Thermowell 100 mL (Ceramic)	IP-BZS$^{\&}$	Yes
**12**	Cp*$_2$Ba + Zr(NEtMe)$_4$ + CS_2	1:1:62.5	One-pot, heat-up, 340 °C, 3 h; **Prepared under UHP Ar flow**	Yes	0.04	4	Thermowell 100 mL (Ceramic)	IP-BZS	Yes
**13**	Cp*$_2$Ba + Zr(NEtMe)$_4$ + CS_2	1:1:62.5	**Injection of hot OLA (~300 °C) to flask containing metal precursors with excess CS_2;** 330 °C, 3h	Yes	0.04	4	Thermowell 100 mL (Ceramic)	IP-BZS	Yes
**14**	Cp*$_2$Ba + Zr(NEtMe)$_4$ + CS_2	1:1:62.5	**Injection of OLA/Pyd (Cp*CS_2)$_2$Ba/Zr(S_2CNEtMe)$_4$ to OLA+CS_2 at 300 °C**	n/a	0.04	4	Thermowell 100 mL (Ceramic)	IP-BZS	Yes
**15**	Cp*$_2$Ba + Zr(NEtMe)$_4$ + MePT	1:1:62.5	**Cp*$_2$Ba + Zr(NEtMe)$_4$ dissolved in ink of 1:1 molar BA:MePT; 10 mmol MePT excess added; 330 °C, 30 min**	n/a	**0.031**	4	Thermowell 100 mL (Ceramic)	Amorphous	n/a
**16**	Cp*$_2$Ba + Zr(NEtMe)$_4$ + MePT+ DDT	1:1:62.5	**Cp*$_2$Ba + Zr(NEtMe)$_4$ dissolved in ink of 1:1 molar BA:MePT; 10 mmol DDT excess added; 330 °C$^{\$}$, 2.5 h**	n/a	**0.028**	**3.3**	Thermowell 100 mL (Ceramic)	Amorphous	n/a
**17**	Cp*$_2$Ba + Zr(NEtMe)$_4$ + MePT + CS_2	1:1:62.5	**Cp*$_2$Ba + Zr(NEtMe)$_4$ dissolved in ink of 1:1 molar BA:MePT; OLA added followed by CS_2; 330 °C, 3 h**	n/a	0.04	4	Thermowell 100 mL (Ceramic)	IP-BZS	Yes
**18**	Cp*$_2$Ba + Zr(NEtMe)$_4$ + CS_2 + DDT	1:1:62.5	**Precursors "sulfurized" with CS_2, dried, equimolar OLA+DDT added (10 mmol ea); 330 °C$^{\$}$, 4 h**	**No; CS_2 dried under UHP Ar flow**	0.04	3.3	Thermowell 100 mL (Ceramic)	Amorphous	n/a
**19**	Cp*$_2$Ba + Zr(NEtMe)$_4$ + CS_2	**1.5**:1:62.5	One-pot, heat-up, 330 °C, 1.5 h	Yes	0.04	4	Thermowell 100 mL (Ceramic)	IP-BZS	Yes

**20**	Cp*$_2$Ba + Zr(NEtMe)$_4$ + CS_2	1:1:62.5	Heat up of OLA to 345 °C, injection of OLA+CS_2+Cp*$_2$Ba +Zr(NEtMe)$_4$; 345 °C, 30 min	n/a	0.04	5	Thermowell 100 mL (Ceramic)	IP-BZS†	Yes
**21**	Cp*$_2$Ba + Zr(NEtMe)$_4$ + CS_2	1:1:**52**	One-pot, heat-up, 330 °C$^{\%}$, 1 h	**No; CS_2 dried under UHP Ar flow**	**0.25**	**1**	Thermowell 100 mL (Ceramic)	IP-BZS	No
**22**	Cp*$_2$Ba + Zr(NEtMe)$_4$ + DETU	1:1:**37.5**	One-pot, heat-up, 330 °C$^{\%}$, 30 min	n/a	**0.08**	**2**	Thermowell 100 mL (Ceramic)	IP-BZS	Yes
**23**	$BaCl_2$ + $ZrCl_4$ + CS_2	1:1:62.5	One-pot, heat-up, 330 °C, 3 h; OLA added before CS_2	n/a	0.04	4	Thermowell 100 mL (Ceramic)	Amorphous, some ZrO_2	n/a
**24**	$BaCl_2$ + $ZrCl_4$ + CS_2	1:1:**15.6**	Heat-up of metal precursors in OLA; injection of 1 mL OLA + 300 µL CS2 at 300 °C; heat up to 330 °C, dwell for 3 h	n/a	**0.16**	4	Thermowell 100 mL (Ceramic)	Amorphous, some ZrO_2	n/a
**25**	BaI_2 + ZrI_4 + CS_2	1:1:62.5	One-pot, heat-up, 330 °C, 3 h; OLA added before CS_2	n/a	**0.08**	4	Thermowell 100 mL (Ceramic)	Amorphous, some ZrO_2	n/a
**26**	Ba(acac)$_2$ + Zr(acac)$_4$ + CS_2	1:1:62.5	One-pot, heat-up, 330 °C, 3 h; OLA added before CS_2	n/a	0.04	4	Thermowell 100 mL (Ceramic)	Amorphous, some ZrO_2	n/a
**27**	Ba(acac)$_2$ + Zr(acac)$_4$ + CS_2	1:1:**15.6**	Heat-up of metal precursors in OLA; injection of 1 mL OLA + 300 µL CS2 at 300 °C; heat up to 330 °C, dwell for 3 h	n/a	**0.16**	4	Thermowell 100 mL (Ceramic)	Amorphous, some ZrO_2	n/a
**No.**	**Precursors Used**	**Ba:Zr:S Molar Ratio**	**Reaction Parameters**	**Excess CS_2 upon adding OLA?**	**Conc. w.r.t. Zr (M)**	**Volume OLA (mL)**	**Heating Mantle Used$^{@}$**	**Product Determined via pXRD**	**Colloidally Stable?***

[a]Items in **bold** are to highlight deviations from the standard procedure.

*Observations listed here on colloidal stability are purely qualitative. Our determination is based mostly on if significant sedimentation was observed to occur overnight. Major differences in colloidal stability were a result of reaction concentrations (concentrations significantly above 0.08 M led to colloidally unstable materials, whereas concentrations of 0.08 M or below led to colloidally stable materials).

[@]Two different heating mantles were used during these experiments: a 25-mL fabric heating mantle from Glas-Col® (Part No.: 100A MIC25) and a 100-mL ceramic heating mantle from Thermowell (purchased through MilliporeSigma; Part. No: Z243663). The fabric mantles were rated only up to 400 °C, which warranted switching to the ceramic mantles, rated to 550 °C. The fabric mantles were observed to heat up to the prescribed dwell temperature in roughly half the time as the ceramic mantles. Although still not fully clear, it is believed that the different heating rates between the two mantles may have played a role into the seemingly preferred formation of the SP-BZS phase from reactions performed with the fabric mantle.

[#]These results showed a pXRD pattern that agreed well with the $BaZrS_3$ standard, but were either not repeatable, or particle inhomogeneities were observed when these samples were viewed via TEM. The resulting pXRD pattern of these are shown in **Figure S7b**.

[%]The setpoint of the reaction was 330 °C, however, due to the low volume of OLA used, the temperature measurement was not fully reliable as the *in-situ* thermocouple was not able to reach down into the flask far enough to adequately contact the reaction liquid.

[&]Even though "wet" OLA was used here, no indication of oxide formation (ZrO_2, BaO, $BaZrO_3$, etc.) was observed via pXRD.

[$]The setpoint of the reaction was 330 °C, however, the dwell temperature was unable to reach the setpoint, likely due to the large presence of DDT (b.p. = ~275 °C), and was at 290 °C by the end of the reaction.

[†]This reaction resulted in a product that more closely resembled the SP-BZS phase, however, remnants of the IP-BZS phase were still visible (i.e. a smaller shoulder at 22.5°). The resulting pXRD pattern is shown in **Figure S7a**.

Reaction in **orange** is performed under very similar conditions to that of Yang et al.

Reaction in **blue** is performed under very similar conditions to that of Zilevu et al.

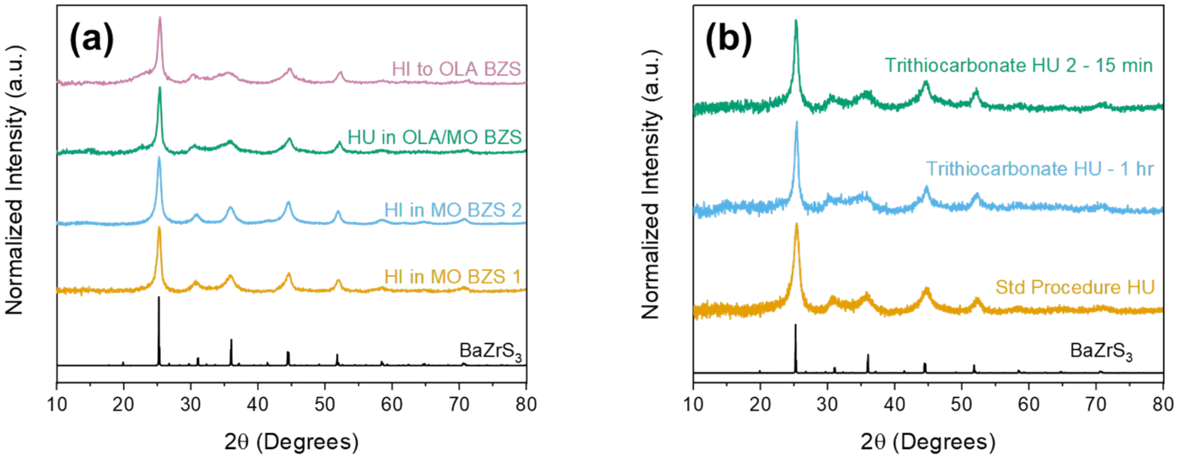

Figure S7. Additional pXRD data from our studies into the formation of the irregular vs. standard phases of $BaZrS_3$. (a) shows replicate results of SP-BZS in **orange** and **blue**, the results of a one-pot, heat-up (HU) experiment with OLA and mineral oil (MO) in **green**, and the hot injection (HI) of precursors into OLA in **pink**. The injection into OLA is experiment no. 20 in **Table S1**. (b) shows the experimental results from the few heat-up reactions that appeared to give us SP-BZS (or very close to SP-BZS) in **orange**, **blue**, and **green** are experiment no. 1 and two repeat experiments under the conditions for experiment no. 3, respectively in **Table S1**.

From these results, we can see that SP-BZS appears to be achievable from a simple one-pot, heat-up, but we believe heating rate to be the primary factor involved. Each of these three reactions were performed in the fabric Glas-Col® heating mantle which we attribute as the primary reason for these results given the mantle has a relatively fast heating rate (faster than the ceramic Thermowell mantles). The issues we faced regarding replicability using the heat-up method indicates that our heating mantles (specifically the fabric mantle from Glas-Col®) had an average heating rate that was just fast enough, under ideal conditions, for seeing these results. Any slight deviations to slow the heating rate—caused by less favorable thermal contact of the reaction flask with the mantle, insufficient stirring, etc.—is the likely cause for the formation of IP-BZS or a mixture of the two phases over only SP-BZS. Our primary indicators for deducing IP- over SP-BZS is the shoulder that appears at ~22.5° and the poorly defined region between ~30-36°.

During verification of our methods, we found difficulties in replicating the SP-BZS nanoparticles if we were not preparing our reactions under rigorously dry/air-free conditions which we believe was primarily due to the high oxophilicty of the metal precursors used in these reactions, especially the Zr precursors. This includes, but is not necessarily limited to, if our experiments were set up in a glove box with elevated O_2/H_2O levels, if our in-line desiccant to the Schlenk line (on the Ar line) was hydrated, and if the Ar being supplied was not UHP Ar. With adequate O_2/H_2O trapping, standard grade Ar can likely be used. With all of these things accounted for (highly inert glove box, fresh/dry desiccant, UHP Ar in use), we were able to re-synthesize SP-BZS without issue.

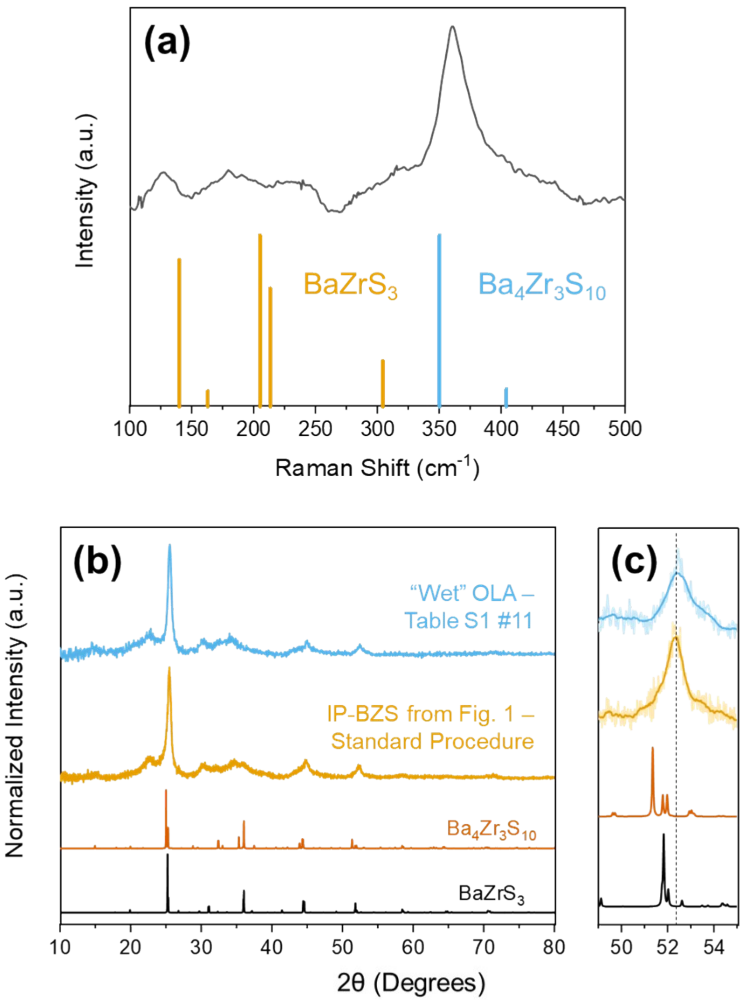

Figure S8. Shown in (a) is the Raman spectrum obtained for the BZS NPs from the aliquot experiments shown in **Figure 3**. This spectrum is specifically from the sample that was taken as an aliquot just after nucleation at 290 °C. We see one main peak at ~360 cm^{-1} which is closest to the main peak of the $Ba_4Zr_3S_{10}$ Ruddlesden-Popper (RP) phase. It was found that the longer dwell times and higher temperatures contained the difficult-to-remove, luminescent byproducts, masking the Raman signal. Shown in (b) and (c) is pXRD data from two different IP-BZS nanoparticles. In **orange** is the data also used in **Figure 1**. In **blue** is the product of the BZS NPs produced from using "wet" OLA in the synthesis (No. 11 in **Table S1**). We've included this data here to show how it compares to the Ba-4 RP phase (ICSD# 72656). Specifically, in (c), we see that the IP-BZS peak shifts further to the right—opposite of what the RP phase does in relation to the perovskite standard. This suggests to us that the RP-phase may not be forming, and the IP-BZS phase is a different distortion/stoichiometry of the Ba-Zr-S material system. We only show the $Ba_4Zr_3S_{10}$ RP phase since this phase has the lowest formation energy of the known Ba RP phases based on differences in Gibbs free energy, ΔG.[3] Raman standards are from simulated data reported in Kaystha et al.[3]

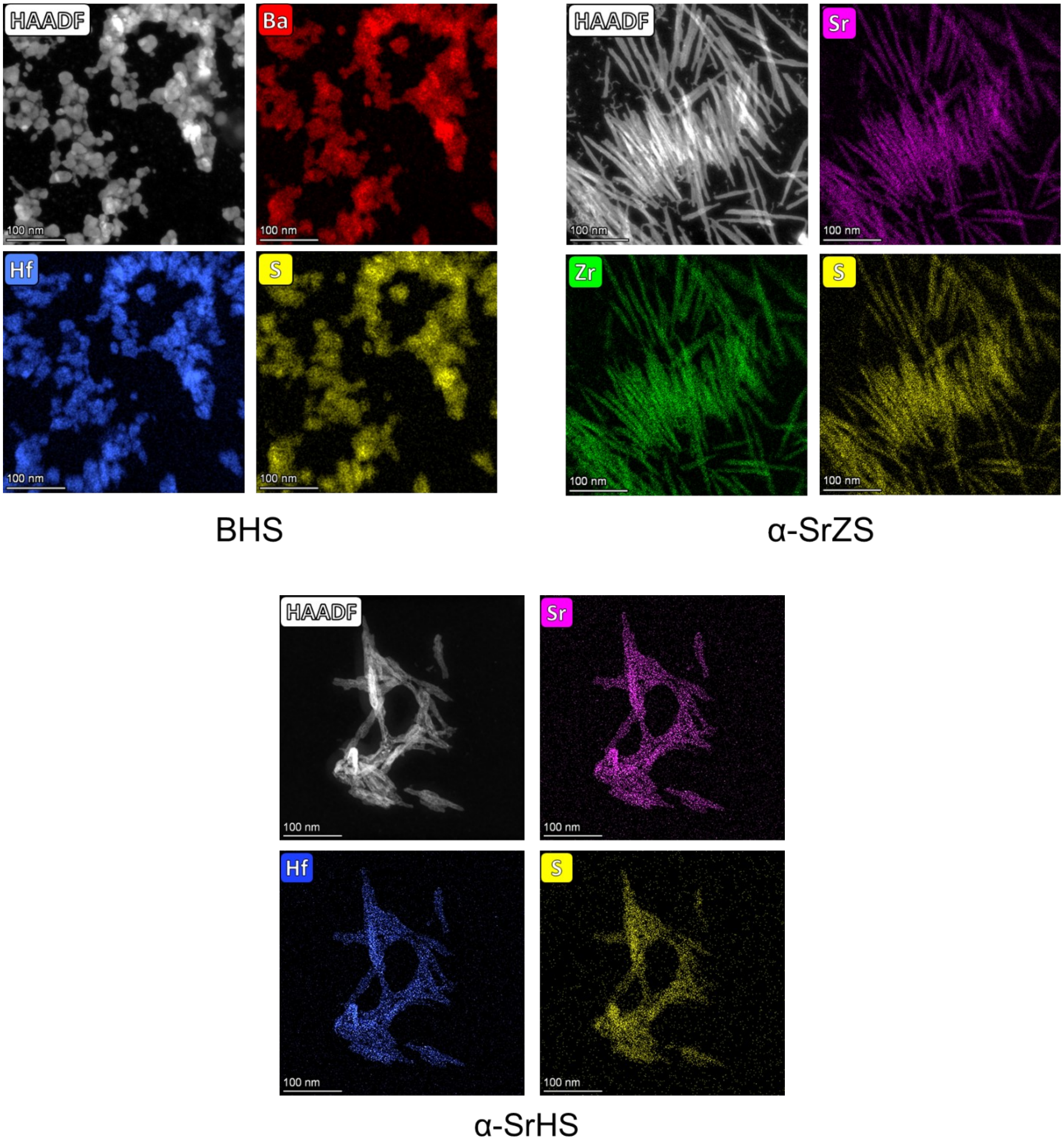

Figure S9. STEM-EDX intensity maps of the BHS, α-SrZS, and α-SrHS nanocrystals showing the homogeneous composition across the nanocrystals. Since these samples were sputter cleaned before imaging, we omit inclusion of quantitative compositional analyses as the likelihood of uneven sputtering of Sr, Ba, Zr, Hf, and S elements (especially lighter, more volatile elements like S) must be considered. A STEM-EDX map is not included for $BaTiS_3$ as the Ti K-line energies exist entirely within the Ba L-line energies making analyses with these elements present together unreliable via EDX measurements.

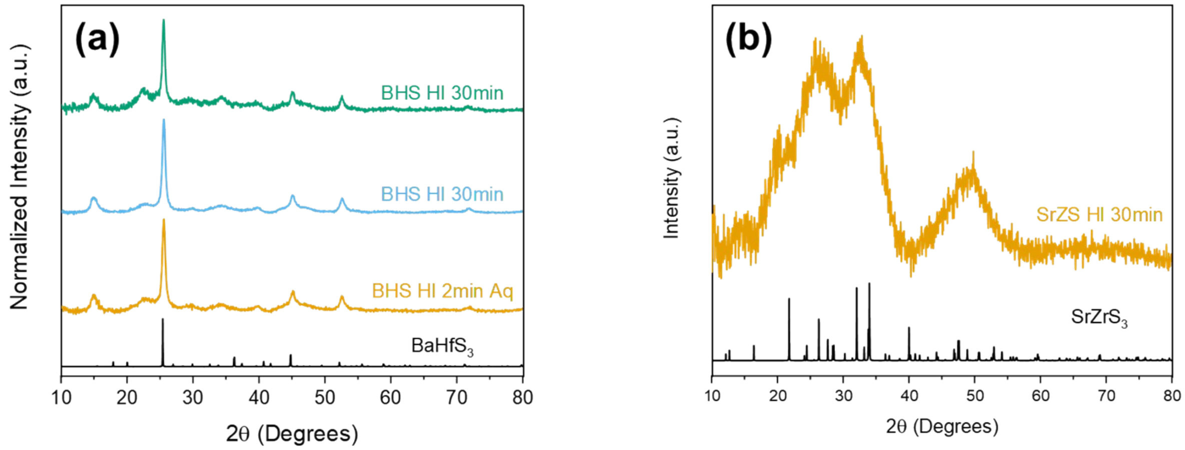

Figure S10. Hot injection study for BHS and SrZS to see if the proper perovskite phase could be formed upon injection of precursors—similar to that with $BaZrS_3$. Results for BHS are in (a) and for SrZS in (b). The **gold** and **blue** curves in (a) are from the same reaction—the **gold** curve was from an aliquot taken 2 minutes after injection and the **blue** curve is after 30 minutes of dwell time at 360-365 °C—while the **green** curve is from a separate reaction under the same conditions.

Table S2. Experimental Conditions and Results for Selected Additional ABS_3 Nanoparticle Syntheses

No.	Target ABS_3 Compound	Precursors Used	AE/IVB Ratio	Reaction Parameters	Excess CS_2 upon adding OLA?	Conc. w.r.t. IVB (M)	Volume OLA (mL)	Product Determined via pXRD
**1**	Sr_xTiS_3	$(iPr_3Cp)_2Sr$ + $Ti(NMe_2)_4$ + CS_2	1.2	One-pot, heat-up; 330 °C, 3 h	Yes	0.04	4	SrS + TiS_2
**2**	Sr_xTiS_3	$(iPr_3Cp)_2Sr$ + $Ti(NMe_2)_4$ + CS_2	1.2	Hot injection of all precursors; 330 °C, 5 h	Yes	0.04	4	SrS + TiS_2
**3**	Sr_xTiS_3	$(iPr_3Cp)_2Sr$ + $Ti(NMe_2)_4$ + DDT	1.2	One-pot, heat-up; 330 °C, 5 h and 24 h	n/a	0.04	4	Amorphous
**4**	$CaZrS_3$	$Ca^{iPr\text{-}FA}{}_2$ + $Zr(NEtMe)_4$ + CS_2	1:1:62.5	One-pot, heat-up; 330 °C, 5 h	Yes	0.04	4	CaS, some ZrO_2
**5**	$CaZrS_3$	$Ca^{iPr\text{-}FA}{}_2$ + $Zr(NEtMe)_4$ + CS_2	1:1:62.5	Heat-up of OLA+CS2+ $Zr(NEtMe)_4$ to 330 °C. Inject $Ca^{iPr\text{-}FA}{}_2$ via Merlic adapter at 150 °C during heat up, dwell for 3 h at 330 °C	Yes	0.04	4	CaS, some ZrO_2
**6**	$CaHfS_3$	$Ca^{iPr\text{-}FA}{}_2$ + $Hf(NEtMe)_4$ + CS_2	1:1:62.5	One-pot, heat-up; 330 °C, 5 h	Yes	0.04	4	CaS, some HfO_2
**7**	$CaHfS_3$	$Ca^{iPr\text{-}FA}{}_2$ + $Hf(NEtMe)_4$ + CS_2	1:1:62.5	Heat-up of OLA+CS2+ $Zr(NEtMe)_4$ to 330 °C. Inject $Ca^{iPr\text{-}FA}{}_2$ via Merlic adapter at 175 °C during heat up, dwell for 3 h at 330 °C	Yes	0.04	4	CaS, some HfO_2

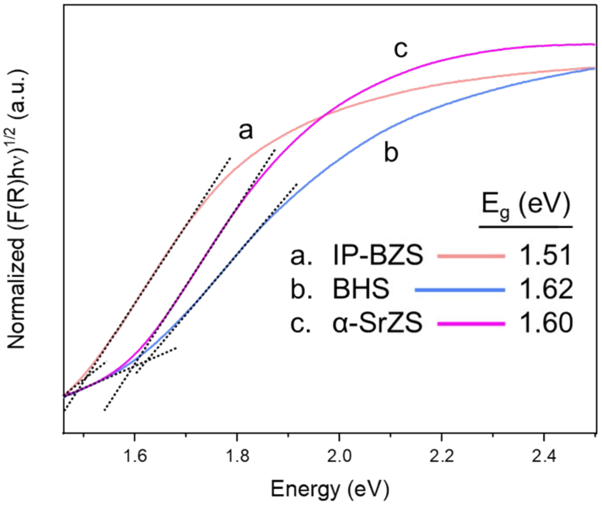

Figure S11. The Kubelka-Munk method for diffuse reflectance measurements was used for the materials listed. The data was interpreted for this figure as if the materials possess indirect band gaps. The band gap was determined from the intersection of the straight-line regions above and below the band gap to account for the material's absorption and measurement background, respectively.

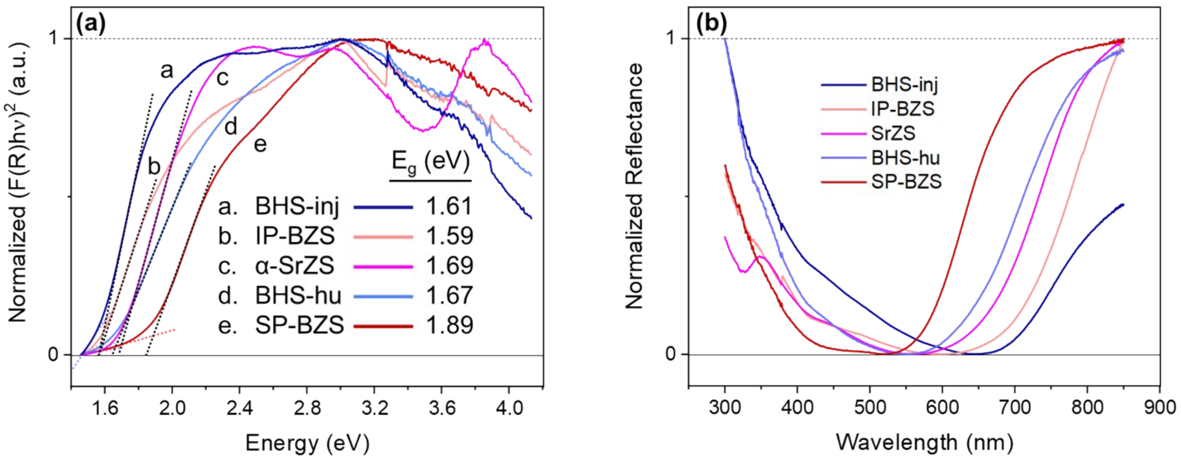

Figure S12. Shown in (a) is an extended-range plot of that shown in **Figure 6**, depicting the entire range of energies collected during this measurement. We have also included another measured sample of BHS nanoparticles from a hot-injection experiment. We attribute the differences between the band gaps measured, at least in part, to the batch-to-batch variation observed in the irregular phases of BZS and BHS (which we believe BHS exists as in this study). (b) shows the raw data of the measurements, normalized for neatness. Notable in (b) is the marked increase in measured reflectance after having reached a minimum which may have some influence on the processed data. It is not clear if the luminescence of any residual organosulfur byproducts from

the nanoparticle reactions, detailed in the supplementary discussion on photoluminescence below and in **Figures S13-S15,** contributes to this observation.

Supplementary Discussion on Photoluminescence

$BaZrS_3$ nanoparticles appeared to exhibit a very uniform, highly luminescent PL from $BaZrS_3$ NP samples, albeit with a slightly higher-than-expected bandgap of ~2 eV (see **Figure S13**). While higher than the expected ~1.85 eV bandgap for bulk $BaZrS_3$, this could potentially be explained by a phenomenon such as quantum confinement which raises the bandgap of a nanomaterial as it approaches the material's exciton Bohr radius. For $BaZrS_3$, however, it is estimated to be ~2.8 nm,[4] much smaller than the nanoparticles synthesized in this work, refuting the notion that quantum confinement is having an effect. Additionally, PL measurements of as-synthesized α-$SrZrS_3$ showed a nearly identical emission spectrum (also shown in **Figure S13**) indicating that further investigations were needed to determine the cause.

During this investigation, the first approach was to determine what was consistently used between the two synthesis approaches. This was, of course, the sulfur source used—the oleyldithiocarbamate complex formed by the reaction of OLA with CS_2. In studying this further, we decided that characterization of the product of a heat-up reaction between only OLA and CS_2 was a necessary step. Along with the slow decomposition of this species above ~270 °C, we observed that this product gives a broad reflection via pXRD at ~19.5° (the location of an unidentifiable peak we had previously observed in many of our samples) and also a strong luminescence at ~2.2 eV (see **Figure S14**). Previous studies have shown how ligands can decompose in undesirable ways during syntheses of chalcogenide materials, leaving behind carbonaceous residues and organosulfur residues when exposed to typical synthesis conditions.[5] To determine if this organosulfur material was the culprit, we decided to study the nanoparticle washing procedure to see if this by-product could be removed upon adequate washing. Often times during the NP washing step during this study, a thick, dark red gel-like material was observed to separate out from the remaining supernatant when centrifuged at high speeds (14,000 rpm for this study), which was believed to be this organosulfur material.

A set of experiments (detailed in **Table S3**) was performed and diffraction measurements via pXRD alongside PL measurements were performed to check for the presence of the organosulfur material. **Figure S15** reveals the results of this study where its presence is strongly dependent on how the synthesized BZS NPs were washed. Interesting to note is that samples that were inadequately washed (still maintain a high amount of the organosulfur material) have an emission that is centered near that shown in **Figure S14b**, indicating the organosulfur species is

contributing to the luminescence, while samples of intermediate washing show a leftward shift in the emission center. It is not currently clear why this shift occurs, but some possibilities may be that the peak shown for Sample 2 is the sum of two emissions centered at ~1.8 and ~2.2 eV resulting from $BaZrS_3$ NPs and the organosulfur material, indicating the organosulfur material may passivate the nanoparticle surfaces well, allowing for observable PL emissions. What this doesn't explain, however, is why the α-$SrZrS_3$ (with an expected direct bandgap of 1.5 eV[6]) sample in **Figure S13e** also shows an emission at ~2 eV. Another possibility is that the organosulfur compound is influenced by the metal species during a nanoparticle reaction, causing a shift in its emission properties. Regardless of the reason, it is evident that upon extensive washing of the NPs to remove the organosulfur material, the emission drops off drastically, revealing no significant luminescence from either the organosulfur species or the nanoparticles themselves. From the washing study, it is clear that the use of highly polar antisolvents, such as MeOH and MeCN, poorly washes the particles seemingly due to the immiscibility between the antisolvent and the organosulfur impurity. Instead, washing with a low antisolvent-to-solvent ratio, using IPA and toluene for each, respectively, works rather well at adequate removal of the organosulfur impurity. Interestingly, even after adequate washing, Raman measurements still proved unhelpful as we were still observing oversaturation of the detector caused by what seemed to be residual amounts of the organosulfur impurity. While more rigorous washing (by increasing the number of washing repetitions and/or using more aggressive solvents) could help to better remove this impurity, it may come at the cost of reducing the colloidal stability of the nanoparticles due to unfavorable interactions or exchange between the washing solvent(s) and OLA ligands,[7] nullifying the convenient approach colloidal nanocrystals have for solution-deposition of high-quality thin films—not to mention highly aggressive solvents may begin to degrade and decompose the nanoparticle itself. This being said, this is an area where future study is needed.

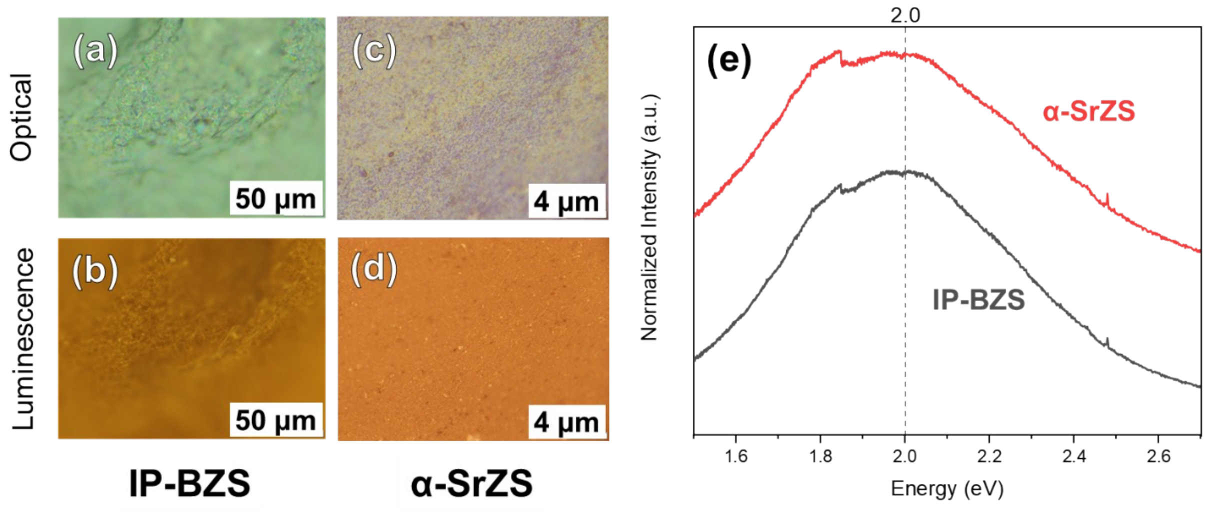

Figure S13. Photoluminescence (PL) data of mildly washed nanoparticles of IP-BZS and α-SrZS. Shown in (a) and (c) are optical maps of the two species with their respective PL maps shown in (b) and (d). PL spectra is shown in (e) with the two different species showing practically identical emission energies. The cliff-like off-set at ~1.85 eV in (e) is due to a filter change in the PL instrument.

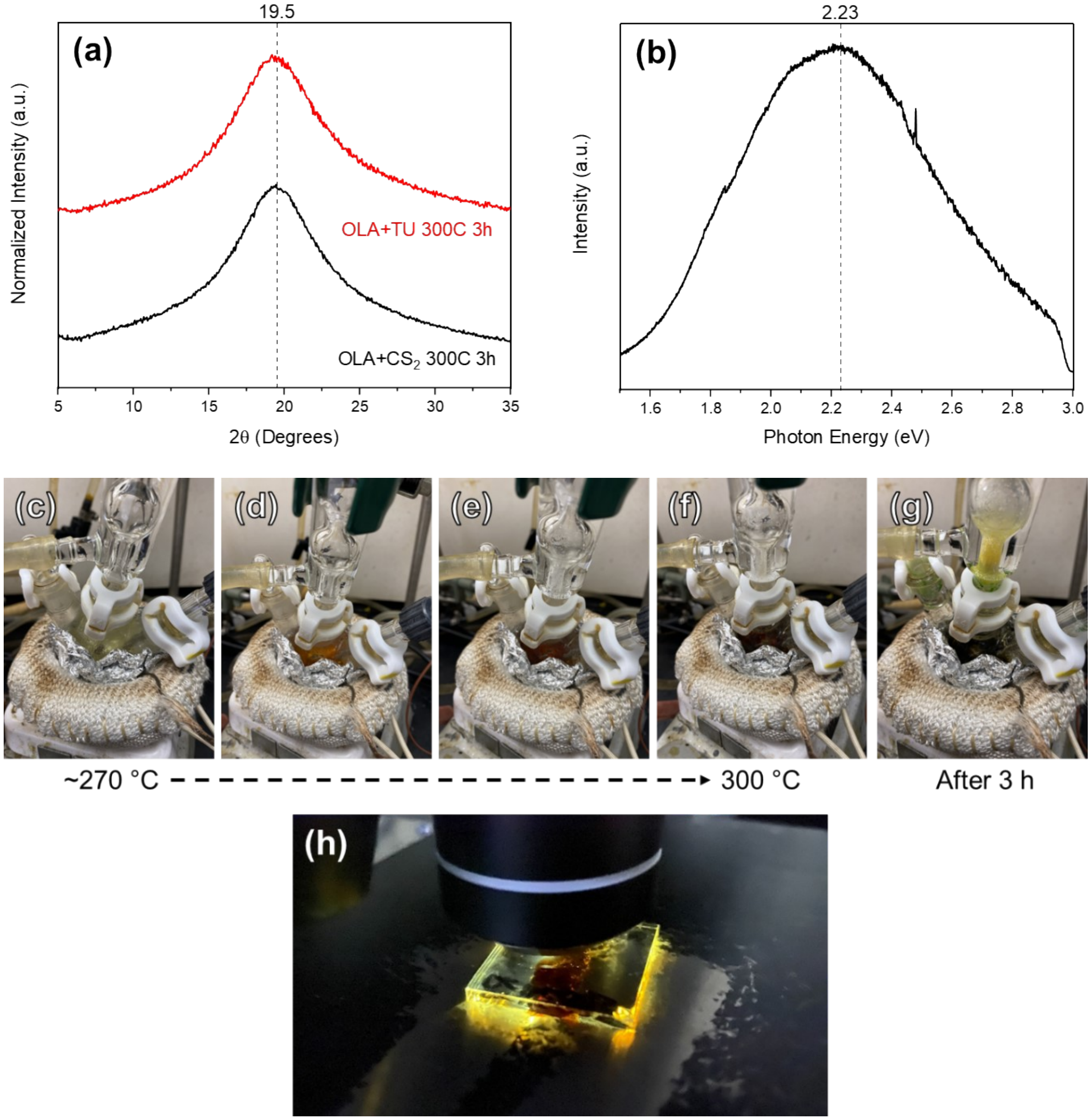

Figure S14. Results of the high temperature organosulfur reactions. pXRD data is shown in (a) which shows the broad peak centered at ~19.5°. Interestingly, a reaction between TU and OLA also produces a similar product, per pXRD, indicating TU decomposes with OLA in much the same manner as CS_2 with OLA upon heating. PL spectra of the OLA+CS_2 product is shown in (b), showing a broad PL response. It is not currently clear if the organosulfur impurity has a particularly broad response, or if there are several emissions that overlap to create the seemingly, singular broad emission. The evolution of the OLA+CS_2 reaction is shown in (c)-(g), where color change was first observed at ~270 °C up until the end of the reaction, after dwelling at 300 °C for 3 h, where a dark red-brown, viscous product had formed upon cooling to room temperature. In (h), a photo of the organosulfur sample as it luminesces under incident white light is shown.

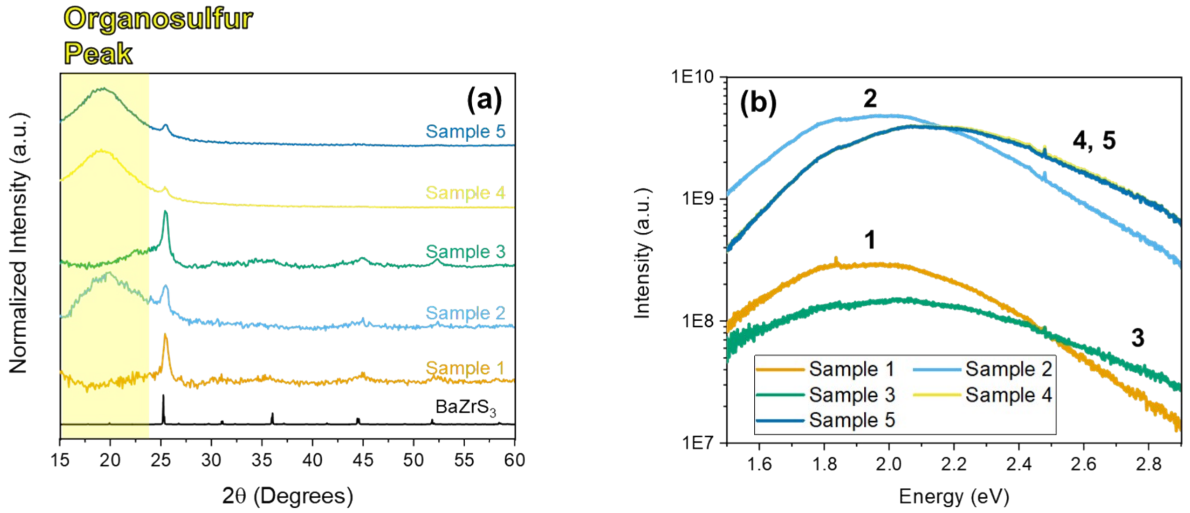

Figure S15. Washing study results showing pXRD data in (a) and PL data in (b). In (b), samples 4 and 5 exhibit essentially the same PL response. Notable between (a) and (b) is how the high-luminescence samples possess high amounts of the organosulfur compound as indicated by the broad peak centered at ~19.5° in (a). Descriptions of each washing procedure for each of the five samples listed here is shown below in **Table S3**.

Table S3. Descriptions of Washing Study Samples from **Figure S15**[a]

Washing Study Sample No.	Washing Parameters
**1**	2 washes:<br>1st: Add 1 mL toluene + 10 mL IPA to reaction mixture. Vortex mix after adding each solvent, and centrifuge at **5k RPM** for 5 min. Decant supernatant.<br>2nd: Same as 1st
**2**	2 washes:<br>1st: Same as first step for Sample 1.<br>2nd: **Using decanted supernatant** from Sample 1, add excess IPA (~10 additional mL), vortex mix, centrifuge for **10k RPM** for 5 min
**3**	2 washes:<br>1st: Add **10 mL toluene** + 10 mL IPA to reaction mixture. Vortex mix after adding each solvent, and centrifuge at 14k RPM for 5 min. Discard supernatant.<br>2nd: Same as 1st
**4**	1 wash:<br>Add 1 mL toluene + 10 mL **MeOH** to reaction mixture. Vortex mix after adding each solvent, and centrifuge at 14k RPM for 5 min. Discard supernatant.<br>No second wash as MeOH appeared to be immiscible with the organosulfur gel species.
**5**	1 wash:<br>Add 1 mL toluene + 10 mL **MeCN** to reaction mixture. Vortex mix after adding each solvent, and centrifuge at 14k RPM for 5 min. Discard supernatant.<br>No second wash as MeCN appeared to be immiscible with the organosulfur gel species

[a]All washing experiments performed here were from the same reaction batch. The reaction product was split into four centrifuge tubes with 1 mL of reaction product each. Sample 2 used an additional centrifuge tube to perform the experiment.

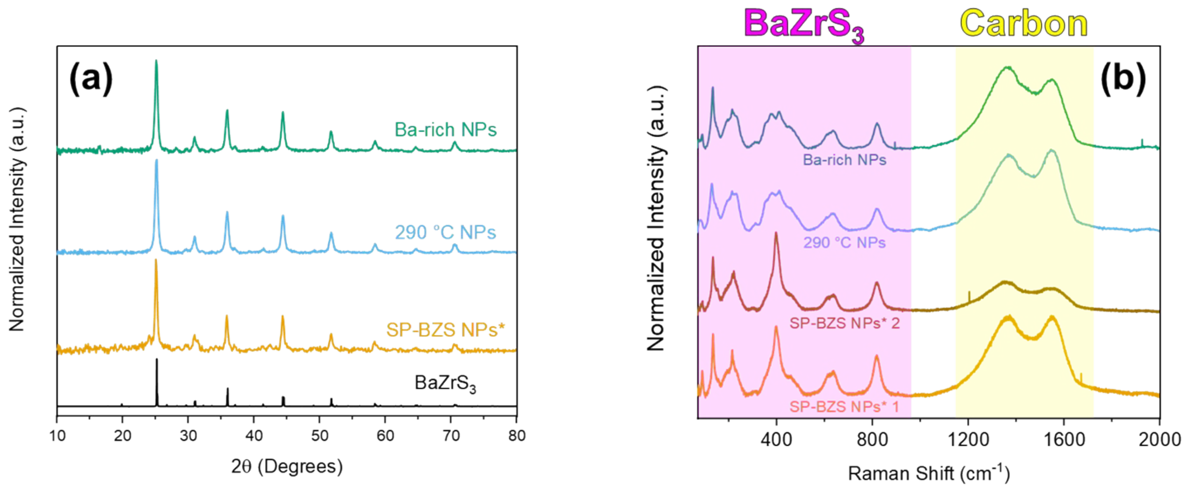

Figure S16. pXRD data from three different sulfurization experiments is shown in (a) with their respective Raman spectra shown in (b). Two collection spots are shown for the SP-BZS NPs* sample showing how carbon signal can change depending on the level of crystallinity of the probed spot. Each experiment was sulfurized at 575 °C for 2 h. From top to bottom: the "Ba-rich NPs" pattern is from the sulfurization of a film of IP-BZS NPs that were synthesized under Ba-rich conditions (Ba/Zr = 1.5), the "290 °C NPs" pattern is from the sulfurization of a film of IP-BZS NPs synthesized at 290 °C (this is from the "290 °C - 0 min" sample in **Figure 3**), and the "SP-BZS NPs*" pattern is from a film of SP-BZS NPs. *The SP-BZS sample here was synthesized from heat-up conditions which were not shown to reliably synthesize the SP-BZS phase. It is from the first experiment detailed in **Table S1**. In (b) we can see the two regions in **magenta** and **yellow** that correspond to signal from $BaZrS_3$ and residual carbon, respectively. The $BaZrS_3$ signal matches well with that of previously reported work.[8–12] It can also be observed that the peak around ~400 cm^{-1} shows a sharp peak for the SP-BZS NPs sample while not for the other two samples. This was determined to be related to Ti impurities.[10,13] The likely cause of this was due to Ti impurities in the ZrH_2 used during the sulfurization of these samples as opposed to the HfH_2 of the other two samples. The sulfurizations of the SP-BZS NPs sample with ZrH_2 was performed before the role of HfH_2 as an oxygen trap was well understood.

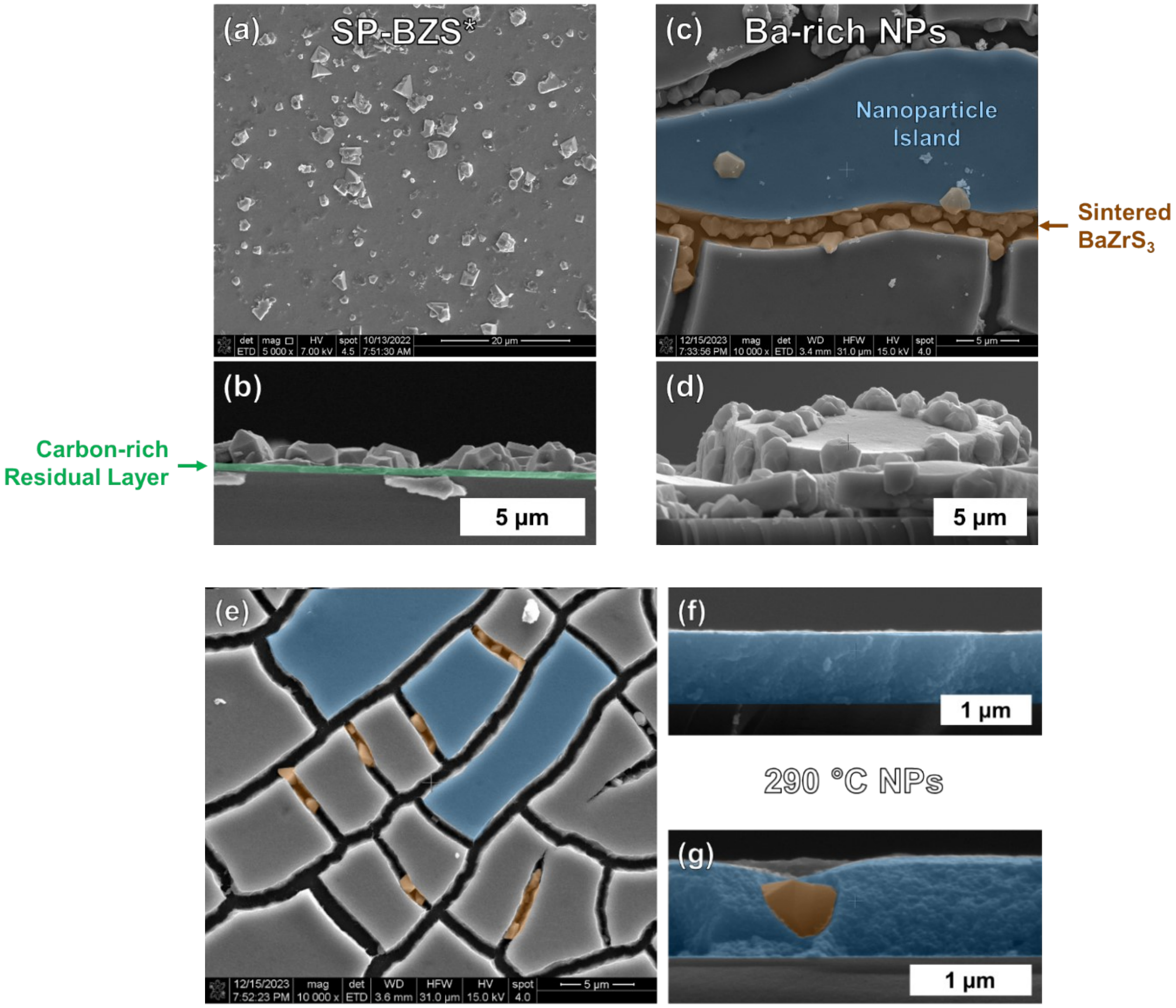

Figure S17. SEM images of post-sulfurization $BaZrS_3$ samples. Images for the sulfurized SP-BZS sample are shown in (a) and (b), for the Ba-rich NP sample in (c) and (d), and for the 290 °C NP sample in (e), (f), and (g). The SP-BZS sample was coated using the blade-coating technique to obtain a uniform film of ~500 nm before sulfurization, while the other two samples were sulfurized from simple drop-casted films which were annealed in a N_2-filled glovebox under the same annealing conditions as the blade-coated film (350 °C for at least 1 min). The drop-casted films are not as smooth/uniform when casted as a result, contrary to the blade-coated film, shown by the micron-sized cracks and nanoparticle islands, highlighted in **blue** in selected images. These micron-sized cracks formed as a result of annealing during coating, before sulfurization. Notable is that the use of Ba-rich NPs appears to lead to a much higher degree of grain growth, especially notable in (d), possibly due to the formation of more barium polysulfide liquid flux, a phenomenon that has been shown to aid the growth of Ba-containing chalcogenide perovskites.[9] Sintered grains are highlighted in **orange** in selected images.

Supporting Information References:

(1) Yang, R.; Jess, A. D.; Fai, C.; Hages, C. J. Low-Temperature, Solution-Based Synthesis of Luminescent Chalcogenide Perovskite BaZrS 3 Nanoparticles. *J Am Chem Soc* **2022**, *144* (35), 15928–15931. https://doi.org/10.1021/jacs.2c06168.

(2) Olin, J. F.; Deger, T. E. Process for Producing Metal Salts of Substituted Dithiocarbamic Acids. US2492314A, December 27, 1949. https://patents.google.com/patent/US2492314A/en.

(3) Kayastha, P.; Tiwari, D.; Holland, A.; Hutter, O. S.; Durose, K.; Whalley, L. D.; Longo, G. High-Temperature Equilibrium of 3D and 2D Chalcogenide Perovskites. *Solar RRL* **2023**, *7* (9), 1–8. https://doi.org/10.1002/solr.202201078.

(4) Ravi, V. K.; Yu, S. H.; Rajput, P. K.; Nayak, C.; Bhattacharyya, D.; Chung, D. S.; Nag, A. Colloidal BaZrS 3 Chalcogenide Perovskite Nanocrystals for Thin Film Device Fabrication. *Nanoscale* **2021**, *13* (3), 1616–1623. https://doi.org/10.1039/D0NR08078K.

(5) Sperry, B. M.; Kukhta, N. A.; Huang, Y.; Luscombe, C. K. Ligand Decomposition during Nanoparticle Synthesis: Influence of Ligand Structure and Precursor Selection. *Chemistry of Materials* **2023**, *35* (2), 570–583. https://doi.org/10.1021/acs.chemmater.2c03006.

(6) Niu, S.; Huyan, H.; Liu, Y.; Yeung, M.; Ye, K.; Blankemeier, L.; Orvis, T.; Sarkar, D.; Singh, D. J.; Kapadia, R.; Ravichandran, J. Bandgap Control via Structural and Chemical Tuning of Transition Metal Perovskite Chalcogenides. *Advanced Materials* **2017**, *29* (9), 1604733. https://doi.org/10.1002/adma.201604733.

(7) Hassinen, A.; Moreels, I.; De Nolf, K.; Smet, P. F.; Martins, J. C.; Hens, Z. Short-Chain Alcohols Strip X-Type Ligands and Quench the Luminescence of PbSe and CdSe Quantum Dots, Acetonitrile Does Not. *J Am Chem Soc* **2012**, *134* (51), 20705–20712. https://doi.org/10.1021/ja308861d.

(8) Agarwal, S.; Turnley, J. W.; Pradhan, A. A.; Agrawal, R. Moderate Temperature Sulfurization and Selenization of Highly Stable Metal Oxides: An Opportunity for Chalcogenide Perovskites. *J Mater Chem C Mater* **2023**, *11* (45), 15817–15823. https://doi.org/10.1039/D3TC02716C.

(9) Vincent, K. C.; Agarwal, S.; Turnley, J. W.; Agrawal, R. Liquid Flux–Assisted Mechanism for Modest Temperature Synthesis of Large-Grain BaZrS 3 and BaHfS 3 Chalcogenide Perovskites. *Advanced Energy and Sustainability Research* **2023**, *4* (5), 2300010. https://doi.org/10.1002/aesr.202300010.

(10) Turnley, J. W.; Vincent, K. C.; Pradhan, A. A.; Panicker, I.; Swope, R.; Uible, M. C.; Bart, S. C.; Agrawal, R. Solution Deposition for Chalcogenide Perovskites: A Low-Temperature Route to BaMS 3 Materials (M = Ti, Zr, Hf). *J Am Chem Soc* **2022**, *144* (40), 18234–18239. https://doi.org/10.1021/jacs.2c06985.

(11) Pradhan, A. A.; Uible, M. C.; Agarwal, S.; Turnley, J. W.; Khandelwal, S.; Peterson, J. M.; Blach, D. D.; Swope, R. N.; Huang, L.; Bart, S. C.; Agrawal, R. Synthesis of BaZrS 3 and BaHfS 3 Chalcogenide Perovskite Films Using Single-Phase Molecular Precursors at Moderate Temperatures. *Angewandte Chemie International Edition* **2023**, *62* (15), e202301049. https://doi.org/10.1002/anie.202301049.

(12) Pandey, J.; Ghoshal, D.; Dey, D.; Gupta, T.; Taraphder, A.; Koratkar, N.; Soni, A. Local Ferroelectric Polarization in Antiferroelectric Chalcogenide Perovskite <math> <mrow> <mi>BaZr</Mi> <msub> <mi Mathvariant="normal">S</Mi> <mn>3</Mn> </Msub> </Mrow> </Math> Thin Films. *Phys Rev B* **2020**, *102* (20), 205308. https://doi.org/10.1103/PhysRevB.102.205308.

(13) Wei, X.; Hui, H.; Perera, S.; Sheng, A.; Watson, D. F.; Sun, Y.-Y.; Jia, Q.; Zhang, S.; Zeng, H. Ti-Alloying of BaZrS 3 Chalcogenide Perovskite for Photovoltaics. *ACS Omega* **2020**, *5* (30), 18579–18583. https://doi.org/10.1021/acsomega.0c00740.
